# The XNA alphabet

**DOI:** 10.1093/nar/gkaf635

**Published:** 2025-07-12

**Authors:** John C Chaput, Martin Egli, Piet Herdewijn

**Affiliations:** Department of Pharmaceutical Sciences, University of California, Irvine, CA 92697-3958, United States; Department of Chemistry, University of California, Irvine, CA 92697-3958, United States; Department of Molecular Biology and Biochemistry, University of California, Irvine, CA 92697-3958, United States; Department of Chemical and Biomolecular Engineering, University of California, Irvine, CA 92697-3958, United States; Department of Biochemistry, School of Medicine, Vanderbilt Ingram Cancer Center, and Vanderbilt Center for Structural Biology, Vanderbilt University, Nashville, TN 37232, United States; Department of Pharmaceutical Sciences, University of California, Irvine, CA 92697-3958, United States; Laboratory of Medicinal Chemistry, KU Leuven, Rega Institute for Medical Research, Leuven 3000, Belgium

## Abstract

Inspired by nature, chemists have spent the last 50 years systematically designing and synthesizing a vast array of sugar-modified nucleic acids, so-called xenonucleic acids (XNAs), collectively forming what we now describe as the XNA alphabet. Within the alphabet, systems can be categorized into two major groups: those capable of interacting with natural nucleic acids and those that do not cross-pair with DNA or RNA. The sugar component of XNAs plays a crucial role in defining their conformational space, which, in turn, influences their hybridization properties and potential applications across biotechnology and synthetic biology. This review provides an overview of sugar-modified XNA systems developed to date as well as the geometric parameters and physicochemical principles that have enhanced our understanding of XNA conformational behavior, particularly in relation to their orthogonality to (i.e. inability to cross-pair with) natural nucleic acids. These insights are essential for developing a more rational approach to key processes such as XNA replication and evolution, ultimately paving the way for applications in areas including synthetic genetics, nucleic acid therapeutics, diagnostics, and nanotechnology.

## Graphical Abstract

**Graphical Abstract:**
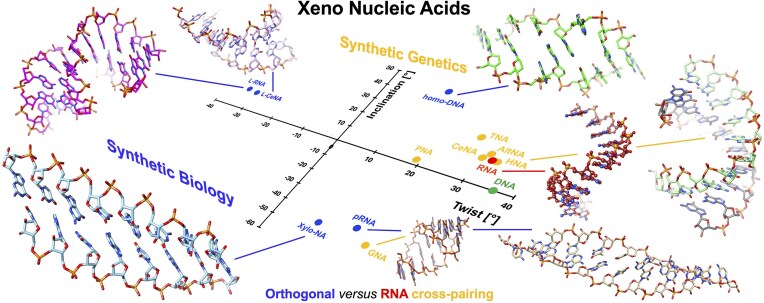

## Introduction

**Figure 1.**
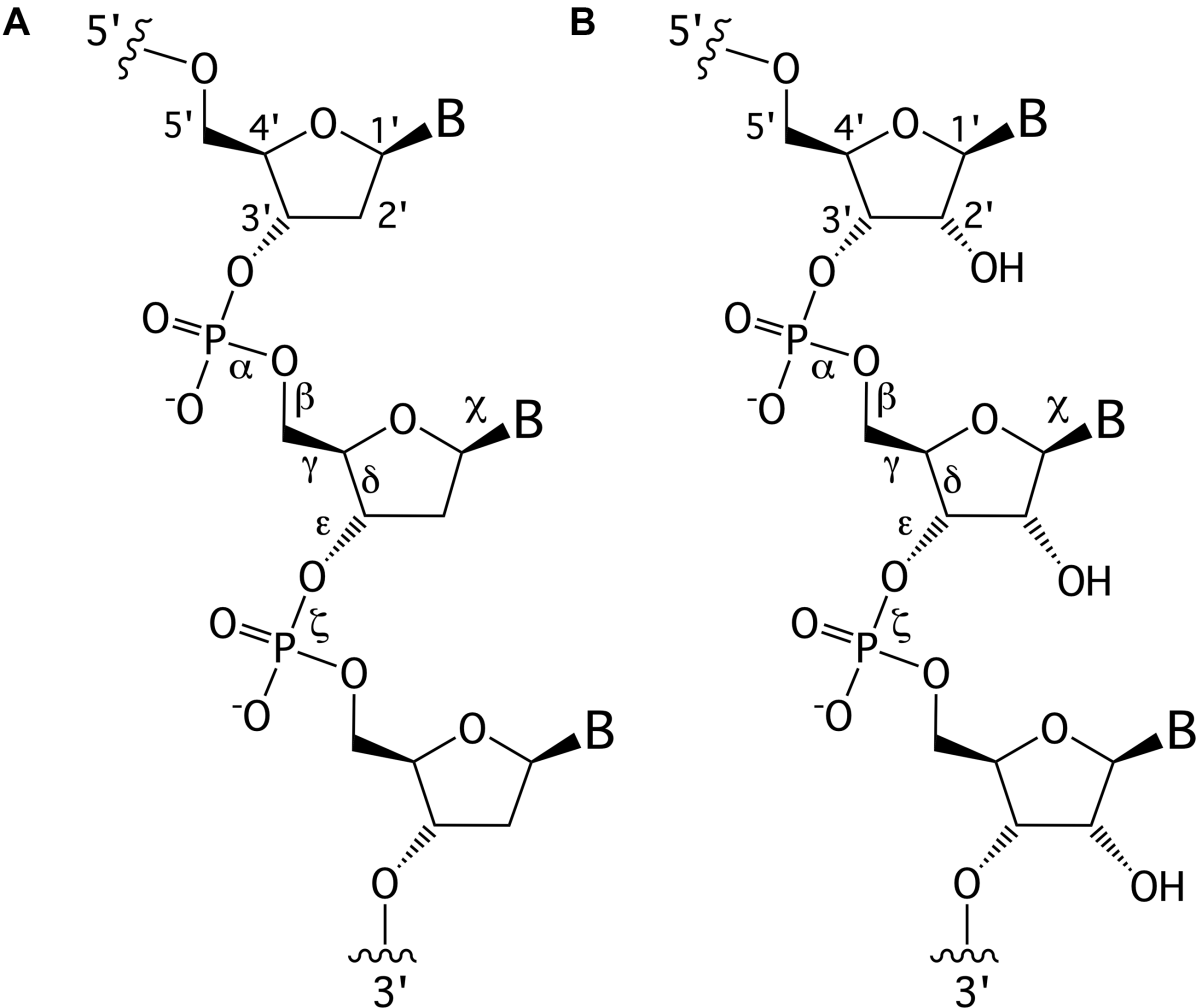
Structures of (**A**) DNA and (**B**) RNA. Backbone and glycosidic torsion angles are labeled..

**Figure 2.**
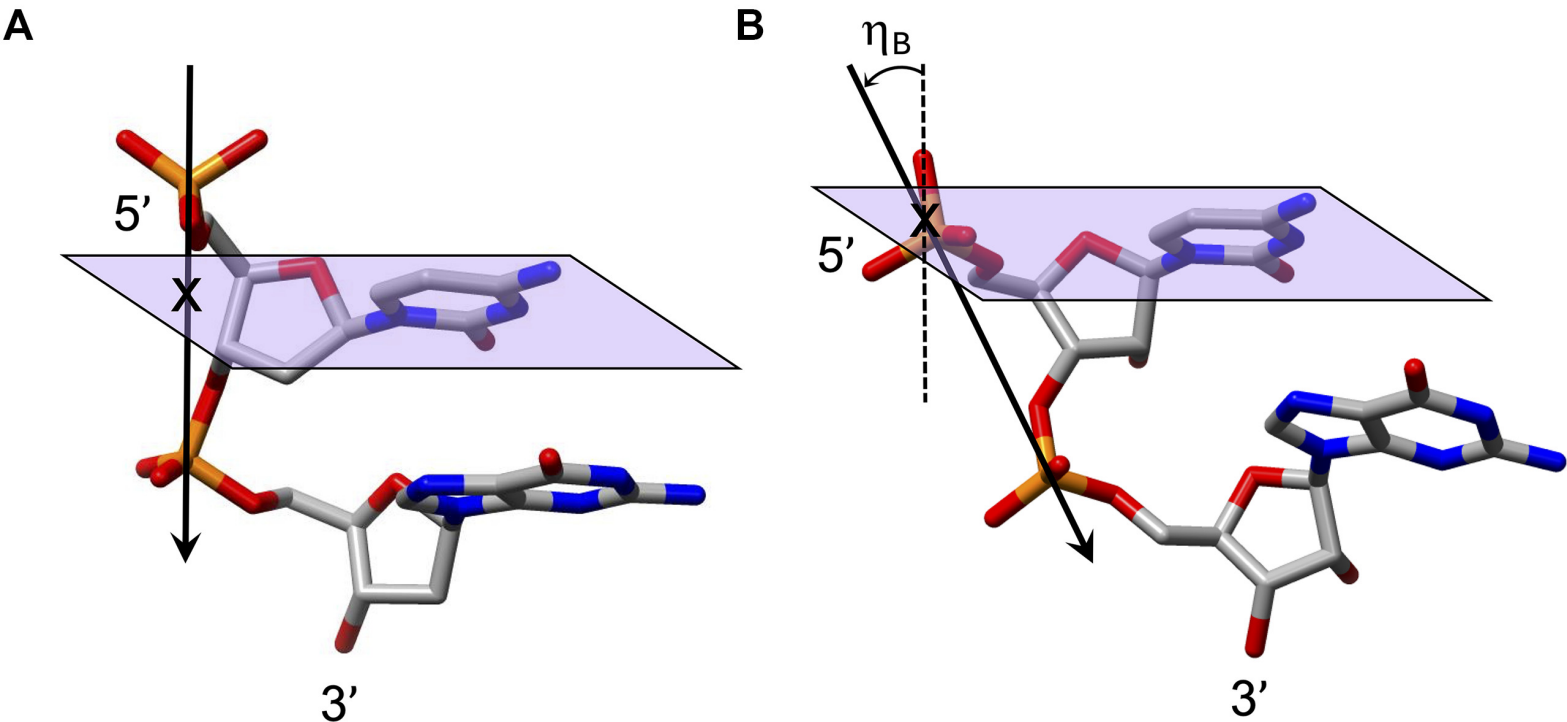
Illustration of the distinct degrees of backbone-base inclination in (**A**) B-form DNA (η_B_ ≈ 0°) and (**B**) A-form RNA (η_B_ ≅ –30°). Vectors connect adjacent phosphorus atoms, a cross marks the point where the P→P vector pierces through the plane defined by cytidine nucleobase atoms, and a dashed line indicates the normal to the plane (panel B).

Natural nucleic acids, 2′-deoxyribonucleic acid (DNA) and ribonucleic acid (RNA), have sugar-phosphate backbones that define their duplex geometry as being helical [1] (Fig. 1). Each helical turn of DNA or RNA consists of 10–12 nucleotide (nt) units, with bases oriented either perpendicular (B- and Z-form DNA) or with a positive inclination (A-form DNA and RNA) relative to the helical axis [2, 3]. Thus, in standard B-DNA, base plane and backbone adopt a normal orientation but in A-RNA, the backbone is negatively inclined relative to the base plane [4] (Fig. 2). Compared to other natural polymers, like proteins and carbohydrates, nucleic acids serve as informational macromolecules, storing and transmitting genetic information through well-established Watson-Crick base pairing rules [5, 6], which are recognized by specialized enzymes.

**Figure 3.**
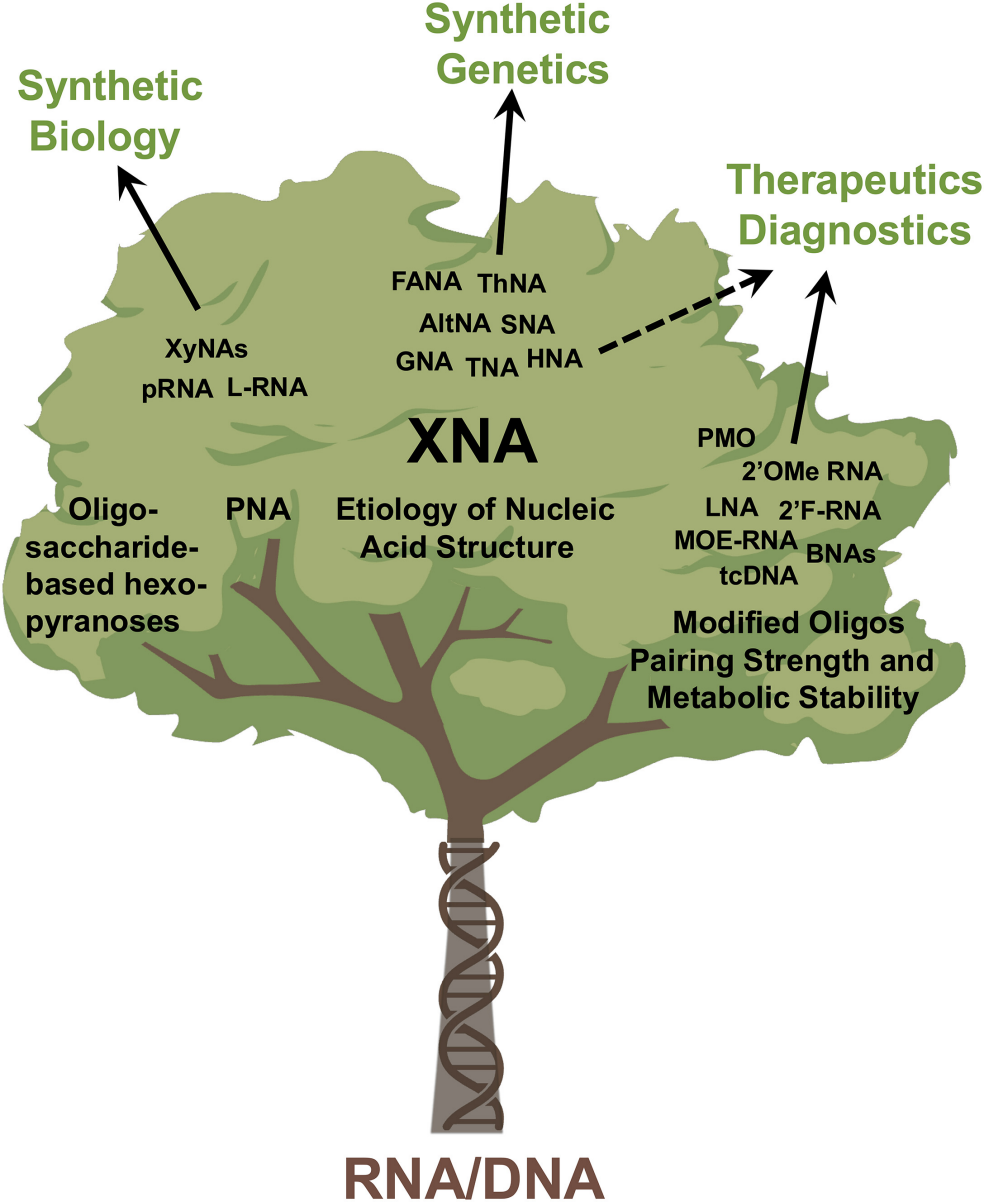
Emerging branches and applications of XNA beyond DNA and RNA. Only selected examples are shown on individual branches and the list is by no means exhaustive.

These fundamental principles of duplex geometry and genetic information storage also apply to artificial genetic systems known as xenonucleic acids (XNAs) [7]. Unlike natural nucleic acids, XNAs feature chemically modified backbones in which the deoxyribose or ribose sugar found in DNA and RNA, respectively, is replaced with an alternative sugar moiety [8, 9]. The rationale for such modifications is to develop synthetic genetic polymers with unique physicochemical properties that can be studied in basic and applied areas of science. In fields such as synthetic biology, materials science, and drug discovery, XNAs are valued for their potential advantages (Fig. 3), including increased hybridization stability, enhanced chemical resistance, and elevated resistance against biological nucleases [10–12]. Fundamental areas, including studies into the origins of life, examine XNAs as progenitor candidates of RNA [13].

Studies into the origin of nucleic acid structure have shown that some XNAs exhibit base-pairing stability comparable to or even superior to natural DNA and RNA [14]. For instance, the melting temperature (T_m_) of a β-D-2′,3′-dideoxyglucopyranosyl nucleic acid (homo-DNA) dodecamer duplex was over 30°C higher than that of its DNA counterpart [15]. This suggests that maximum pairing strength alone was unlikely to be the primary factor in nature’s selection of RNA as the foundation of life's genetic system [16]. Instead, accuracy and uniformity of base pairing in a biologically relevant environment were likely more critical factors.

Most XNA applications developed to date require the faithful recognition of XNA by natural DNA and RNA [17–19]. For XNAs to functionally interact with DNA and RNA, they must be capable of forming helical structures resembling natural antiparallel Watson-Crick duplexes [8]. Researchers have explored four main strategies for engineering XNAs that can hybridize with natural nucleic acids:

- Chemical modification of natural nucleic acids to enhance hybridization strength and enzymatic stability [8], leading to the discovery of locked nucleic acid (LNA) [20], 2′-*O*-(2-methoxyethyl)-RNA (MOE-RNA) [21, 22], and 2′-deoxy-2′-fluoroarabino nucleic acid (FANA) [23, 24].
- Biomimetic approaches using oligopeptides as structural models, inspired by the helical properties of α-helices in proteins. Although α-helices differ geometrically from nucleic acid helices, modifications have enabled the development of peptide nucleic acids (PNAs), which can form stable duplexes with DNA and RNA [25]. A particular characteristic of PNA is that it is charge neutral, which allows it to invade double-stranded (ds) DNA [26].
- Carbohydrate-based designs using oligosaccharides as structural templates. While natural polysaccharides like amylose adopt helical conformations, their geometries typically do not align with DNA/RNA [27]. However, systematic studies of carbohydrate modified nucleic acids led to the discovery of hexitol nucleic acid (HNA), which maintains a helical structure and base-pairing compatibility with DNA and RNA [28].
- Prebiotic chemistry-inspired designs, exploring alternative nucleic acid backbones that could have arisen in a pre-RNA world. This approach led to the discovery of threose nucleic acid (TNA), which can form stable antiparallel duplexes with DNA and RNA despite its backbone being one atom shorter per repeating unit [29].

This review focuses on sugar-modified XNAs and provides a tabulated summary of most of the XNAs synthesized to date and investigated to various degrees with regards to pairing preference and stability. A majority of such XNAs retains hybridization compatibility with natural nucleic acids—highlighting their potential in genetic applications. In this context, synthetic genetics refers to engineered nucleic acids capable of interacting with DNA and RNA to modulate their functions. In contrast, synthetic biology, particularly from a chemical perspective, aims to develop orthogonal genetic systems that operate independently of natural nucleic acids. To this end, researchers have also designed sugar-modified, non-cross-pairing XNAs such as pyranosyl-RNA (pRNA) [30], xylonucleic acid (XyNA) [31], and L-RNA [32]. These systems serve diverse purposes, from exploring the origins of life (e.g. pRNA) to designing nucleic acids with enhanced enzymatic stability for aptamer applications (e.g. L-RNA) and materials science. In the context of synthetic biology and L-RNA/-DNA, some researchers have warned of potentially overlooked risks related to the creation of mirror life. They have called for a broader discussion of the potential creation of lifeforms completely based on mirror-image biological molecules such as DNA and RNA composed of “left-handed nucleotides” and proteins composed of “right-handed” amino acids [33].

In this Critical Perspective and Review, we discuss the geometric parameters including sugar conformation, helicity, helical rise and twist, backbone-base inclination and biophysical features that form the basis for an XNA’s ability to cross-pair with the natural nucleic acids or, alternatively, form a stable orthogonal pairing system of potential interest in the pursuit of synthetic biology. Examples of particular XNAs either of the RNA/DNA cross-pairing type or constituting an orthogonal system, are examined in the contexts of pairing stability and 3D structure. Further, the review discusses the state-of-the-art of XNA polymerase development and selected applications of XNAs in the discovery and development of oligonucleotide therapeutics.

## DNA—the most famous XNA

The primary difference between DNA and RNA is the absence of a hydroxyl group at the 2′-position of the sugar, a fundamental distinction in molecular design (Fig. 1). RNA is considered the first nucleic acid polymer to support life due to its ability to both catalyze chemical reactions (phenotype) and store genetic information (genotype). However, its chemical instability limits RNA genomes in extant life to RNA viruses. Removing the 2′-hydroxyl group results in a more chemically stable polymer that is better suited for storing and transferring genetic information. This transition is supported by considerations of life's origin and present-day cellular biochemistry. Notably, prebiotic synthesis of ribose is more feasible than that of deoxyribose, and modern cells synthesize DNA building blocks from RNA precursors [34].

**Figure 4.**
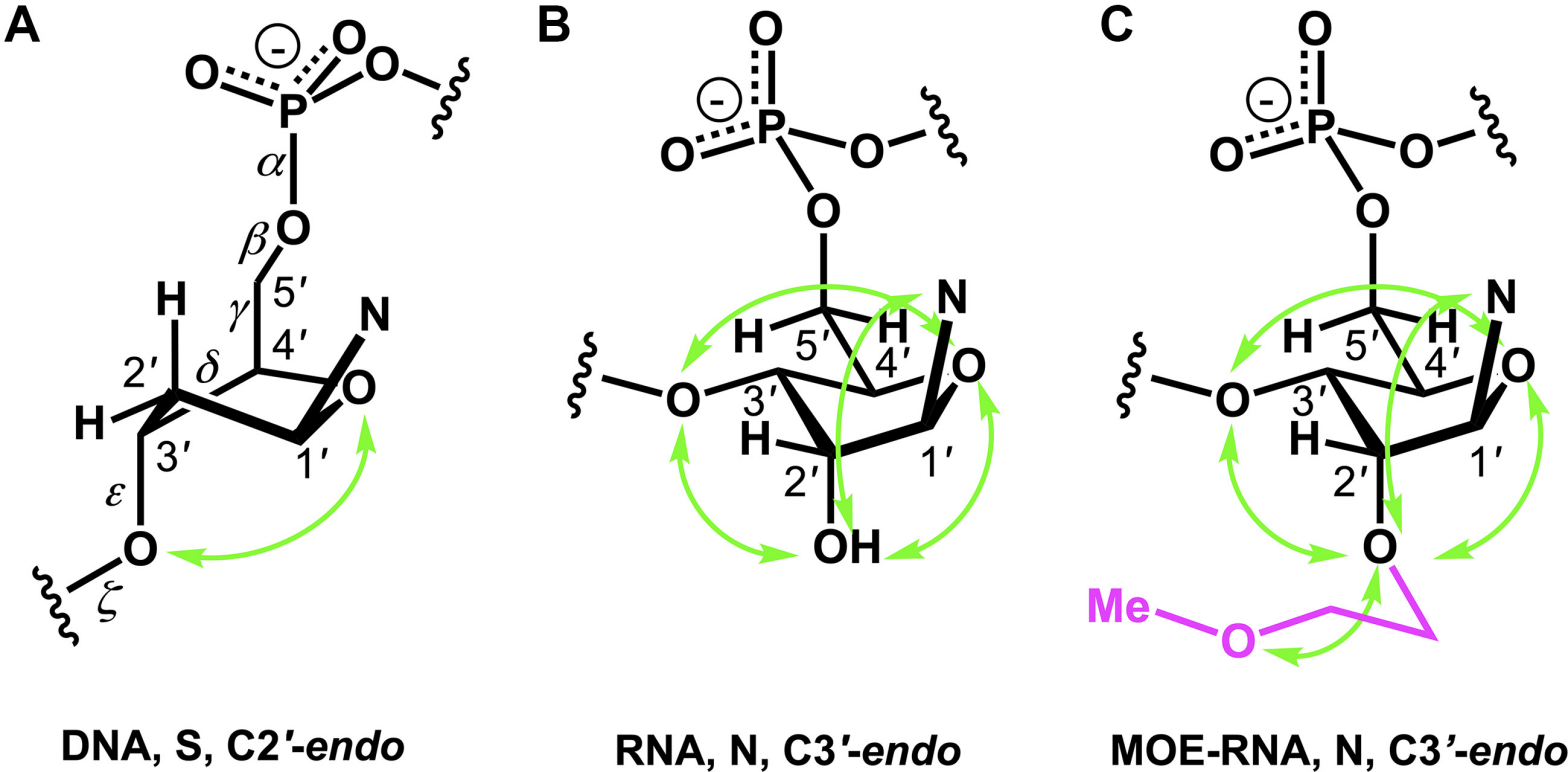
Gauche effects indicated by green arrows in the sugar rings of (**A**) 2′-deoxyribonucleotides (one), (**B**) ribonucleotides (four), and (**C**) 2′-*O*-(2-methoxyethyl)-RNA (MOE-RNA) (five).

Thus, DNA can be regarded as the first XNA, designed by nature specifically for stable information storage with reduced catalytic activity. Nature further refined DNA by replacing RNA’s uracil with thymine. (i) The removal of the 2′-hydroxyl group enhances chemical stability under basic conditions but reduces stability in acidic environments, as well as the polymer's conformational diversity and catalytic potential. This occurs due to the loss of gauche effects (Fig. 4), the electron-withdrawing influence of the hydroxyl group, and the absence of anchimeric assistance in reactions like hydrolysis [35]. (ii) The substitution of thymine with uracil also decreases the acidity of the base moiety (pKa of thymine in thymidine ∼9.8 versus uracil in uridine ∼9.2), suggesting that uracil in RNA may more readily facilitate catalytic activity through deprotonation [36]l. (iii) Cytosine deamination in DNA results in uracil which, if not repaired before replication, leads to a G:C to A:T transition mutation. Repair of U in DNA proceeds via a base-excision pathway initiated by uracil DNA glycosylase [37]. (iv) Due to its function to store genetic information, DNA has a longer lifespan than RNA in a cell. Therefore it is important that DNA be more resistant to photochemical mutations than RNA, which further explains the use of thymine in DNA instead of uracil [38]. (v) Additionally, thymine reduces mismatch formation, improving DNA’s ability to faithfully replicate its genetic information, which was essential at life's origin. (vi) The methyl group in thymine also stabilizes the DNA duplex through base stacking, though its evolutionary significance remains unclear. (vii) The 5-methyl group, always present in in DNA but occurring in RNA only as a modification (m5U), may also have been introduced as a factor to contribute to hydrophobic interactions with protein side chains, and influence groove widths and flexibility of dsDNA for similar reasons (i.e. groove recognition by DNA metabolic enzymes) [39].

## RNA—functional role in biology

RNA holds a unique position among biopolymers due to its distinct physicochemical properties, which are largely attributed to the presence of a ribofuranose sugar in its repeating nucleotide unit. In RNA, the sugar moiety consists of four oxygen atoms, one anomeric nitrogen atom, and five carbon atoms. These atoms are arranged such that each heteroatom is part of at least one X-C-C-Y system, where conformational preferences are governed by the gauche effect.

In ribose (ribonucleotides), four types of gauche effects occur within the five-membered ring, whereas in 2′-deoxyribose (2′-deoxyribonucleotides), only one gauche effect contributes to the pseudorotational equilibrium of the sugar ring (Fig. 4A and B). A simplification is made here regarding the fifth gauche effect (O5'-C5'-C4'-O4'), which has minimal influence on sugar conformation.

The gauche effect is stronger than the anomeric effect, and in 2′-deoxyribonucleotides (DNA), these effects counteract each other, leading the DNA sugar to adopt a South (S-type) conformation [40]. In contrast, in TNA (Table 1), two gauche effects (O2'-C2'-C1'-O4' and O3'-C3'-C4'-O4') and one anomeric effect (O2'-C2'-C1'-N) act in the same direction, stabilizing the sugar in an N-type C4'-exo conformation [41–43]. The role of the gauche effect in nucleic acid preorganization is further illustrated by 2′-*O*-(2-methoxyethyl)-RNA (MOE-RNA) [21, 22, 44, 45], where the 2′-*O*-(2-methoxyethyl) moiety undergoes preorganization thanks to an additional gauche effect in the substituent (Fig. 4C), thereby enhancing duplex stability together with a water molecule bound between O2', O3', and the outer oxygen of the MOE substituent.

**Table 1.** The XNA alphabet

Name	Abbreviation	Structure	Citation
A			
Acyclic Phosphonate Backbone	*R*-ZNA	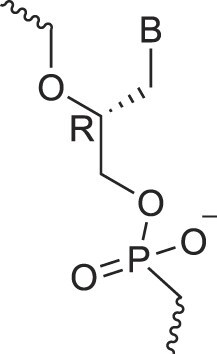	[65]
	*S*-ZNA	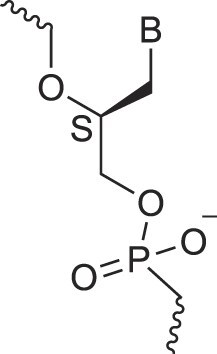	[65]
4′-Alkoxy-2′-deoxy nucleic acids	4′-AlkoxNA	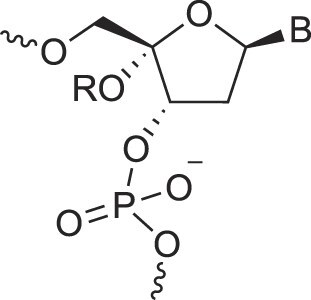	[66]
Alkynyl-2′-deoxy nucleic acids	2′-AlkNA	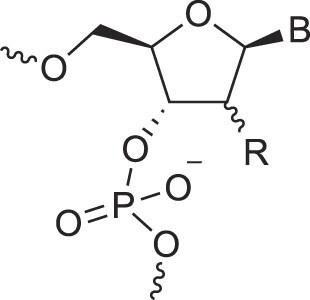	[67]
	4′-AlkNA	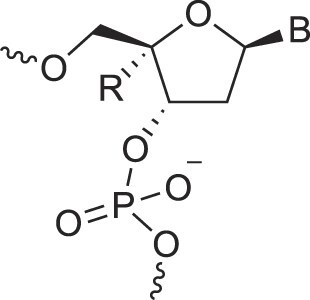	[68]
	5′-AlkNA	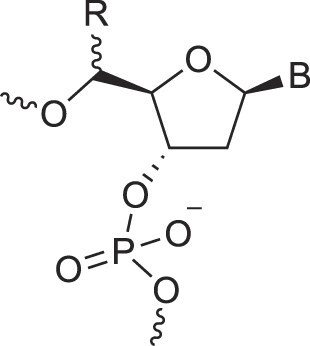	[69]
Arabino nucleic acids	AraNA	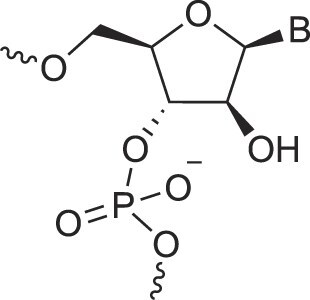	[70]
D-*β*-Altritol nucleic acids	AltNA	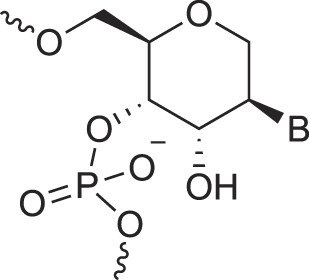	[71]
D-*β*-Allo-nucleic acids	AlloNA	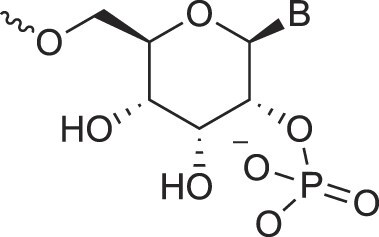	[14]
D-*β*-Altro-nucleic acids	AltroNA	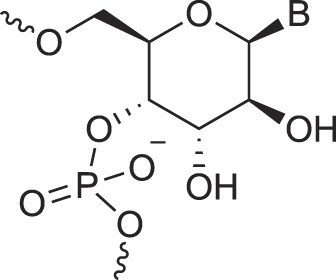	[14]
3′-Arafluoro hexitol nucleic acids	Ara-FHNA	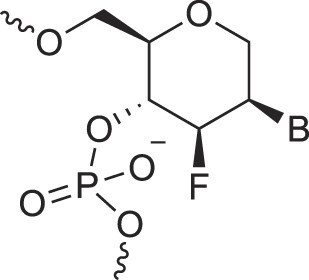	[72]
2′-*O*-Alkylated nucleic acids	2′-*O*-AlkylNA	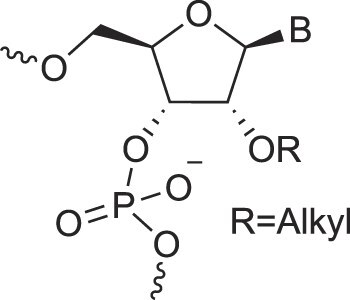	[21]
Aminopropyl nucleic acids	*R*-APNA	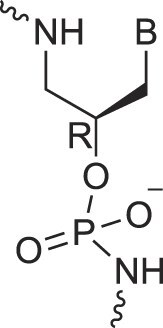	[73]
	*S*-APNA	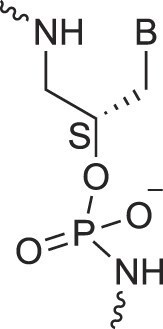	[73]
2′-Amido nucleic acids	2′-AmidoNA	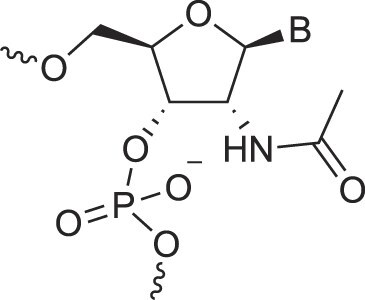	[74]
Apio nucleic acids	ApioNA	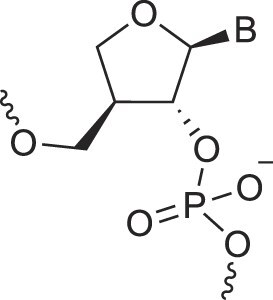	[75]
2′-Amino deoxynucleic acids	NH_2_-RNA	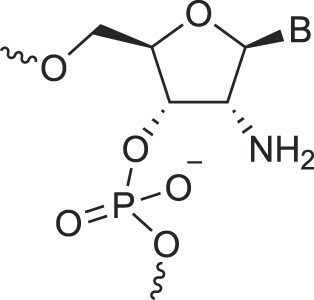	[76]
2′-Azido deoxynucleic acids	N_3_-RNA	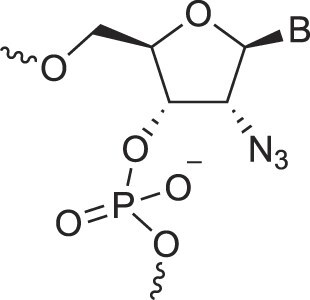	[76]
Amido-bridged nucleic acids	AmNA	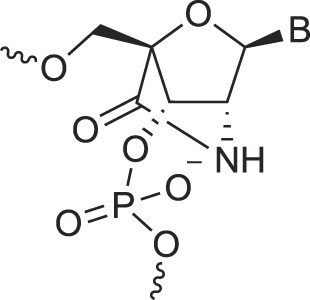	[77]
B			
BicycloDNA	BcDNA	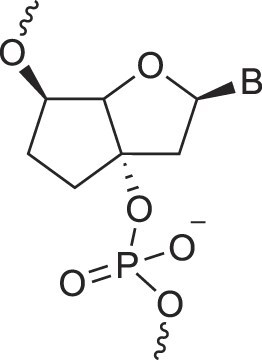	[78]
	Bc[3.2.1]DNA	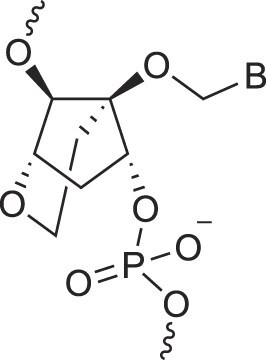	[79]
	Bc[3.2.1]amide-DNA	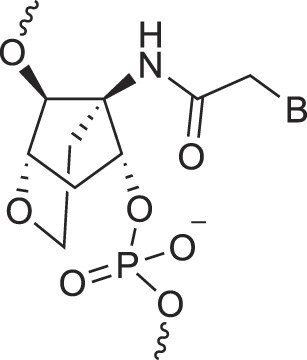	[80]
	Bc[4.3.0]DNA	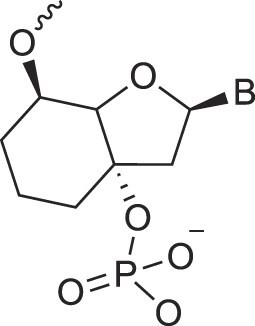	[81]
	2′,3′-BcNA	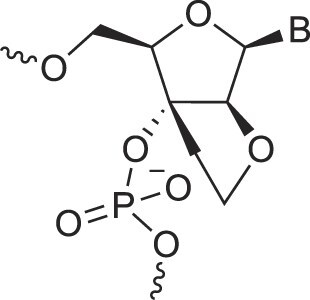	[82]
Butyl nucleic acids	BuNA	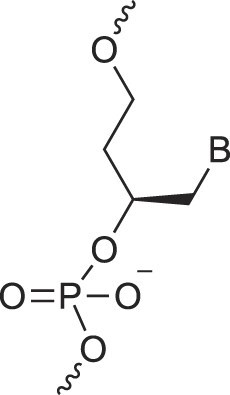	[83]
C			
Carbocyclic DNA	carDNA	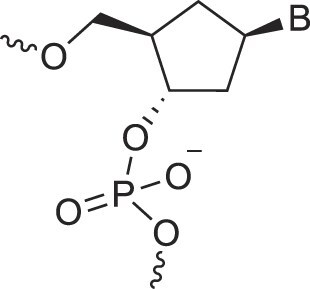	[84]
Carbocyclic RNA	carRNA	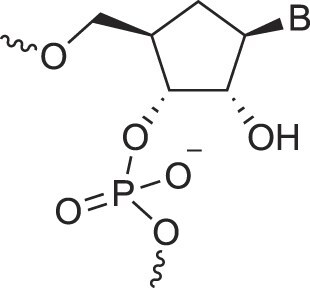	[84]
Cyclohexanyl nucleic acids	CNA	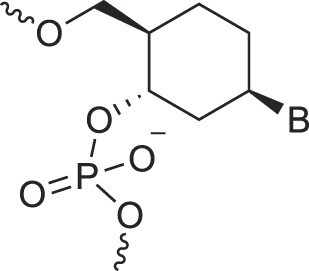	[85]
D-*ribo*-Cyclohexanyl nucleic acids	*r*-CNA	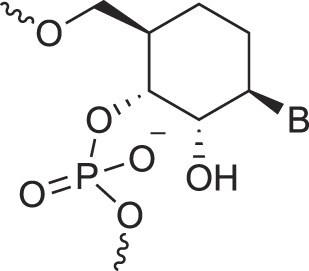	[86]
Cyclohexenyl nucleic acids	D-CeNA	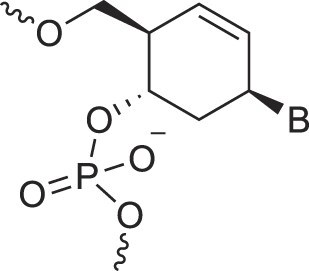	[87]
	L-CeNA	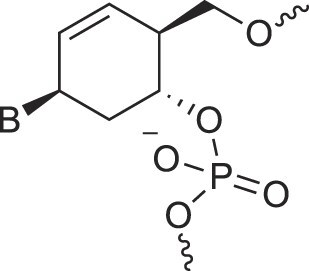	[88]
Carbamate-linked nucleic acids	CarbamateNA	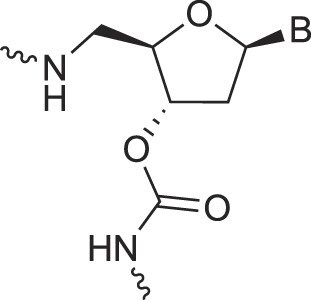	[89]
D			
Deoxyribose nucleic acids	*β*-D-DNA (Natural)	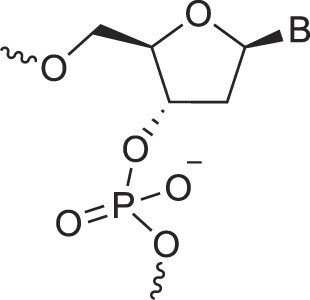	
	*α*-D-DNA	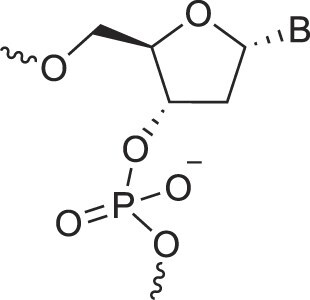	[90]
	*β*-L-DNA (mirror image DNA)	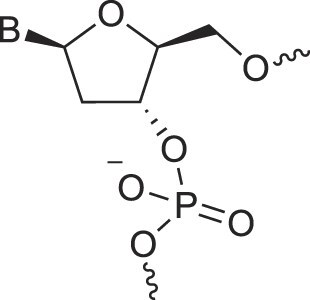	[91]
Di-*O*-Methylated altropyranoside nucleic acids	DMANA	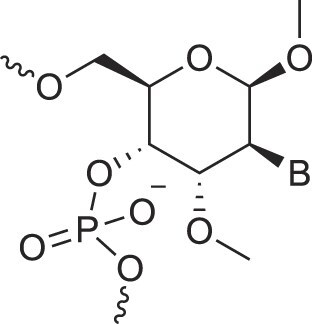	[92]
Double coding nucleic acids	DcDNA	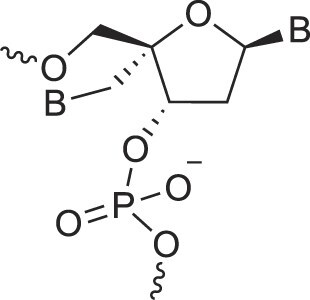	[93]
Disubstituted DNA	2′,4′-DFNA	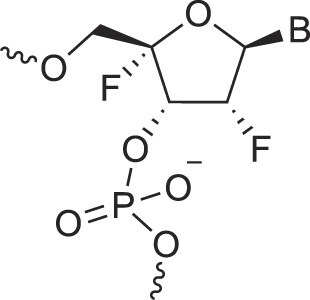	[94]
	2′F,4′-OMe NA	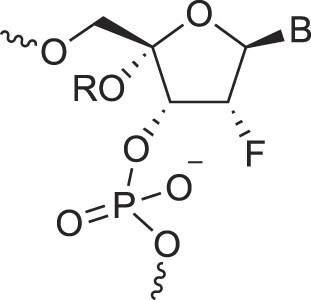	[95]
	2′OMe,4′F NA	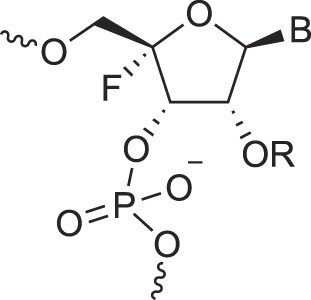	[96]
	2′,4′-diOMe NA	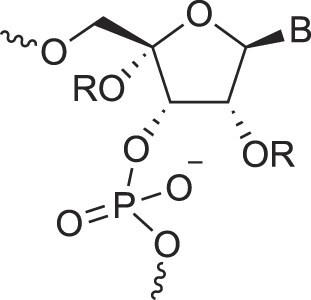	[96]
2,4-Dihydroxycyclohexyl nucleic acids	2,4-DHCNA	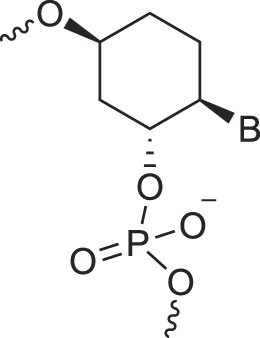	[97]
Double-headed nucleic acids	2′-DhNA	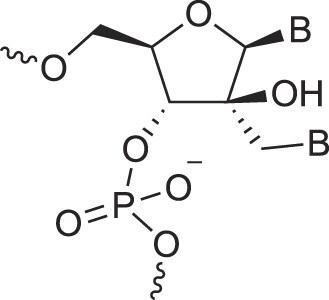	[98]
Diethanol amide nucleic acids	DEANA	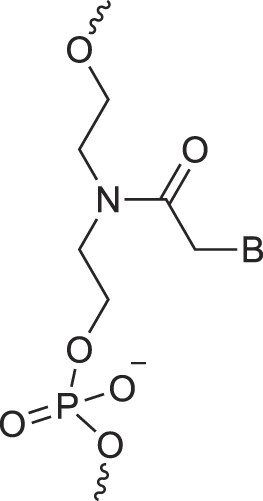	[99]
E			
Ethinyl nucleic acids	ENA	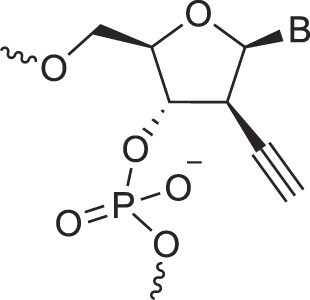	[100]
Ethylene bridged nucleic acids	EBNA	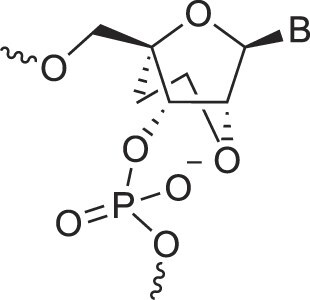	[101]
Extended nucleic acids	ExNA	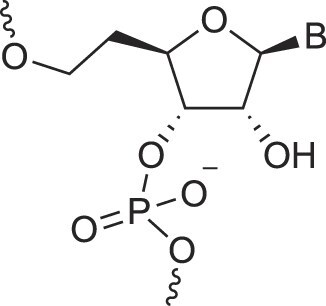	[102]
F			
2′-Fluoro arabino nucleic acids	FANA	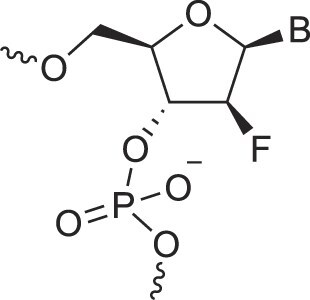	[103]
Flexible nucleic acids	FNA	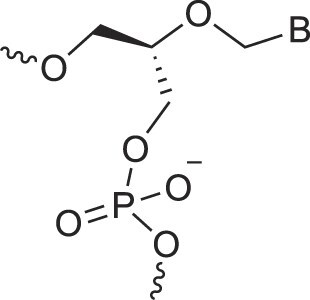	[104]
6′-Fluoro[4.3.0]bicyclo nucleic acid	6′-F-bc[4.3.0]DNA	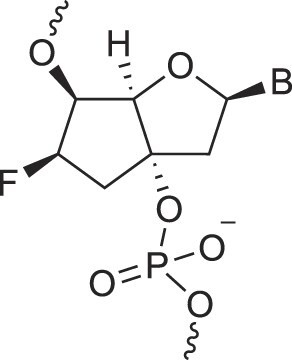	[105]
3′-Fluoro hexitol nucleic acids	FHNA	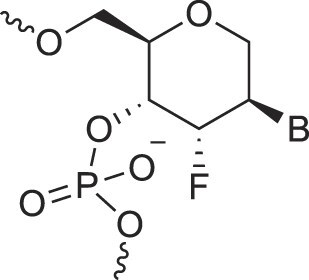	[72]
	FMHNA	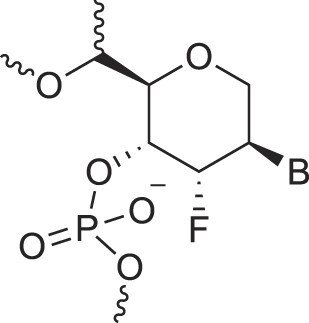	[106]
Fluoro cyclohexenyl nucleic acids	F-h	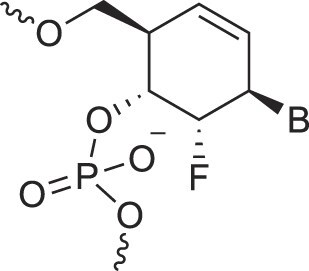	[87]
2′-Formamido nucleic acids	2′-Formamido NA	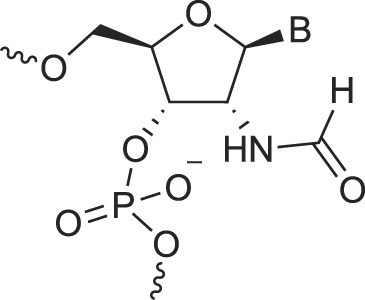	[107]
2′-Fluoro nucleic acids	F-RNA	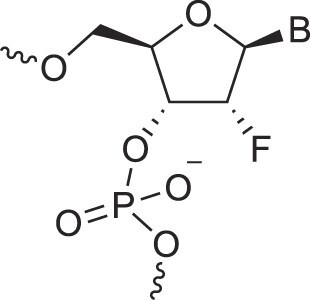	[108]
Ferrocene nucleic acids	FeNA	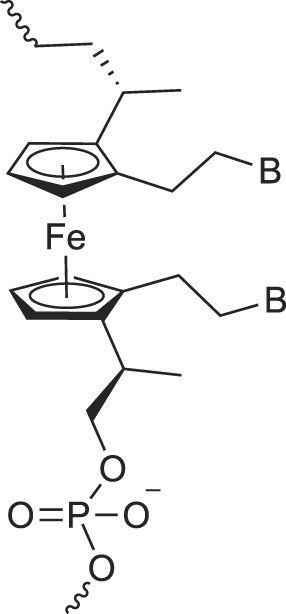	[109]
G			
Glycerol nucleic acids	GNA	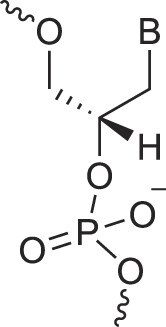	[110]
D-*β*-Gluco nucleic acids	GlucoNA	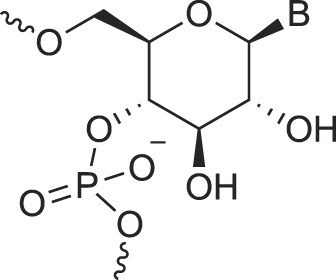	[14]
Glycol carbamate nucleic acids	*R*-GCNA	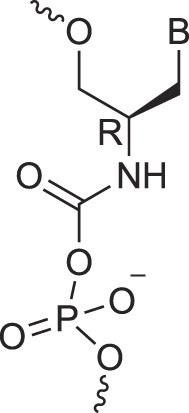	[111]
	*S*-GCNA	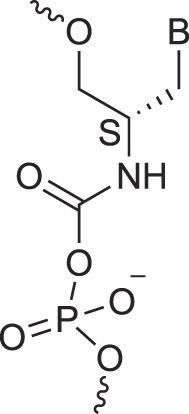	[111]
Glucosamino nucleic acids	3′,6′-GANA	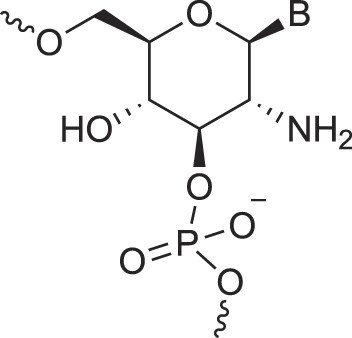	[112]
	4′,6′-GANA	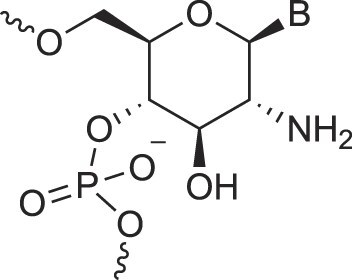	[112]
Guanidine linked nucleic acids	GuanidineDNA	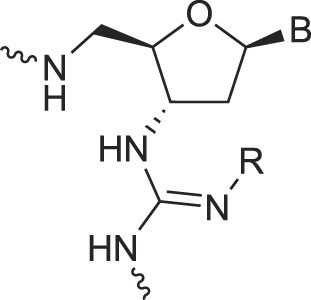	[113]
H			
Hexitol nucleic acids	*α*-L-HNA	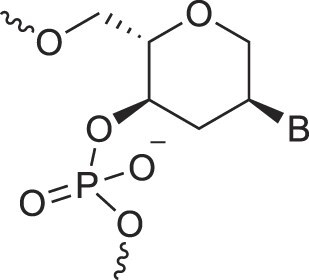	[114]
	*α*-D-HNA	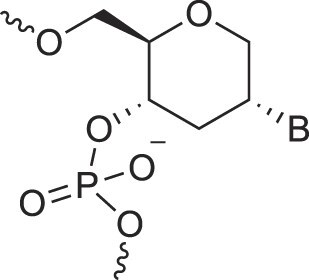	[115]
	*β*-L-HNA	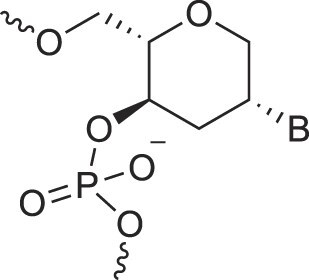	[116]
	*β*-D-HNA	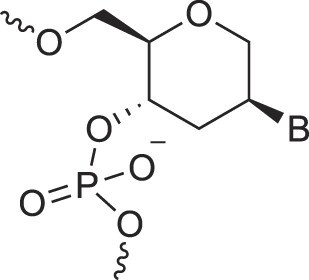	[117]
Hydroxy*-N-*acetylprolinol nucleic acids	Hydroxy*-N-*AcProNA	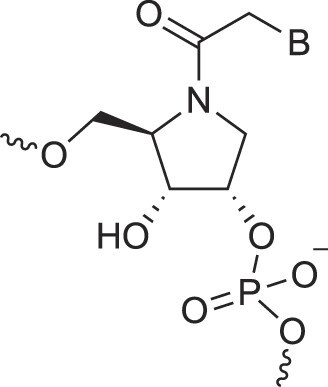	[118]
Homo-N-deoxyribose nucleic acids	*β*-D-Homo-DNA	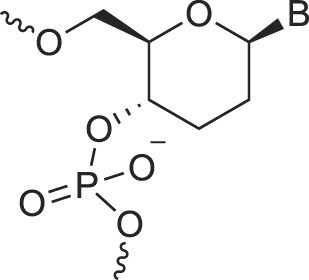	[119]
	*β*-L-Homo-DNA	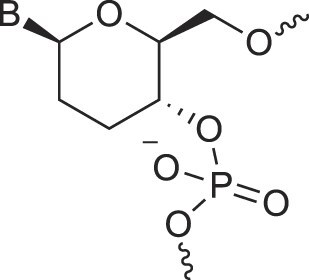	[120]
	*α-*D*-*Homo-DNA	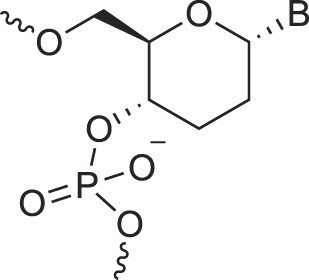	[121]
	1′-homoDNA	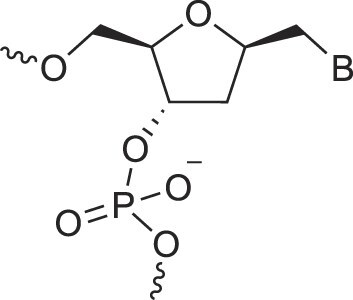	[122]
5′-Hydroxyphosphonate-linked nucleic acids	5′-hpDNA	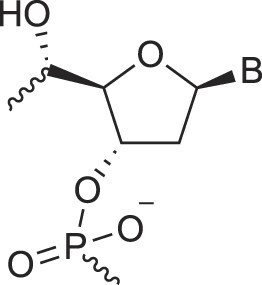	[123]
3′-Hydroxymethyl – aldopentopyranose nucleic acids	3′-hmAPPNA	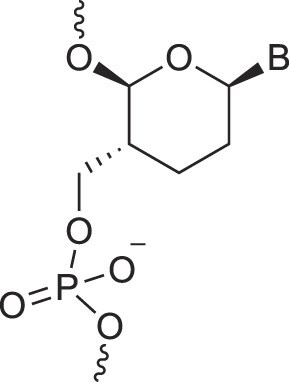	[124]
I			
Intercalating nucleic acids	INA	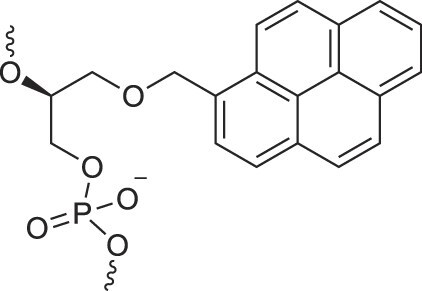	[125]
Isobicyclo-DNA	IsoBcDNA	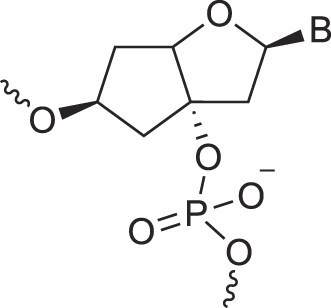	[126]
Iso-glycerol nucleic acids	IsoGNA	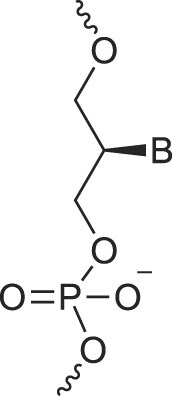	[127]
J, K			
L			
Locked nucleic acids	LNA	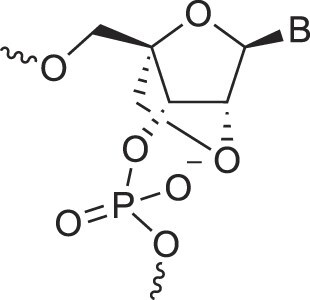	[20]
*α-*Locked NA	*α-*LNA	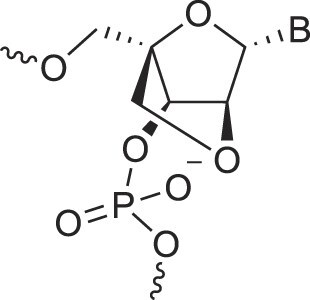	[128]
Methylene-carbocyclo Locked NA	Methylene-cLNA	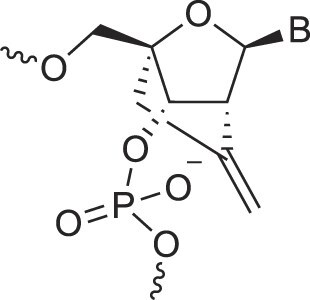	[129]
2′-*O*-Methoxyethyl Locked NA	cMOENA	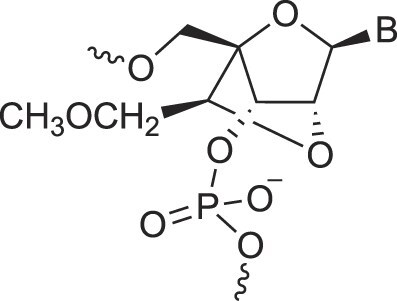	[130]
Cyclic 2′-*O*-Ethyl Locked NA	cEt BNA	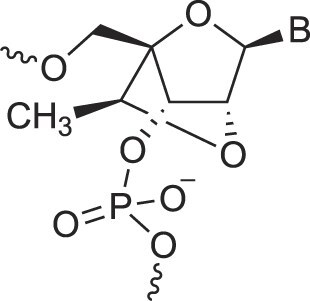	[131]
Lyxo nucleic acids	2′,4′-LyxoNA	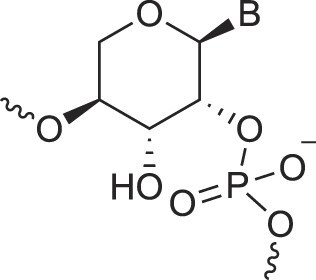	[14]
	3′,4′-LyxoNA	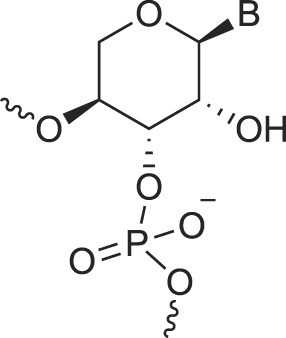	[14]
M			
Morpholino nucleic acids	MorphNA (PMO)	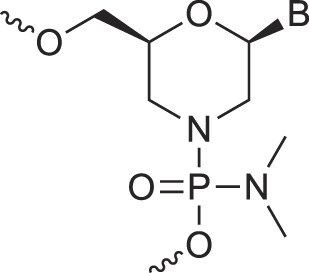	[132]
Mannitol nucleic acids	ManNA	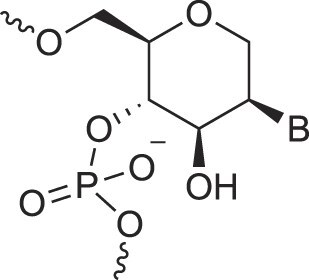	[133]
3′-*O*-Methylated ANA	MANA	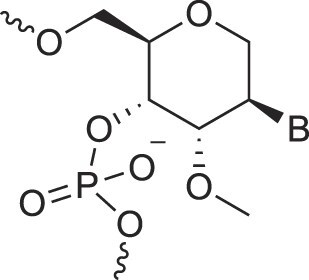	[134]
2′,5′-DNA	MetaDNA	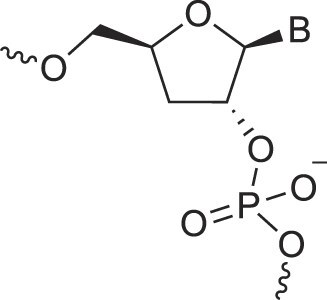	[135]
2′,5′-RNA	MetaRNA	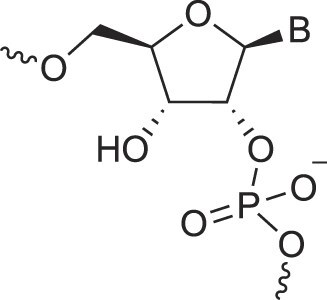	[136]
2′-*O*-Methoxyethyl nucleic acids	MOENA	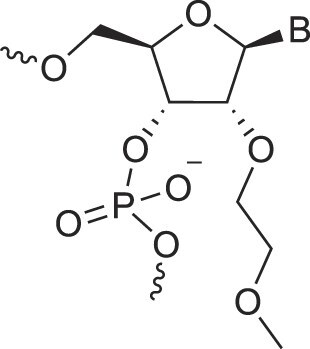	[21]
N			
North Methanocarba DNA	NMDNA	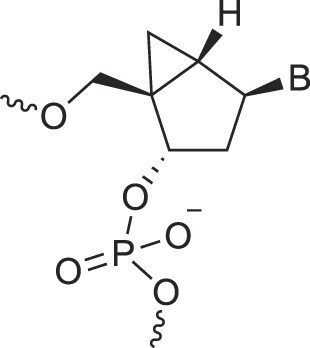	[137]
O			
Oxepane nucleic acids	OxNA	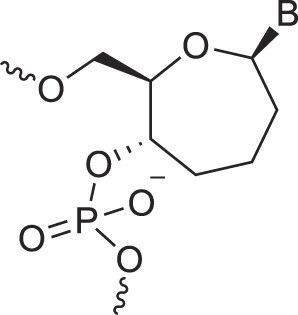	[138]
P			
Peptide nucleic acids	PNA	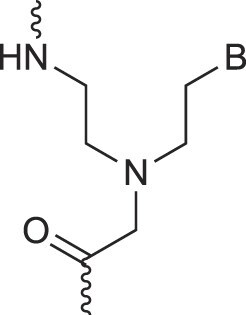	[139]
Phosphoramidate nucleic acids	N3'→N5'-DNA	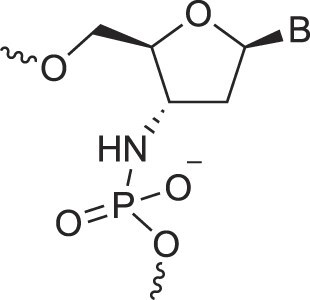	[46]
Polycarbamate nucleic acids	PCNA	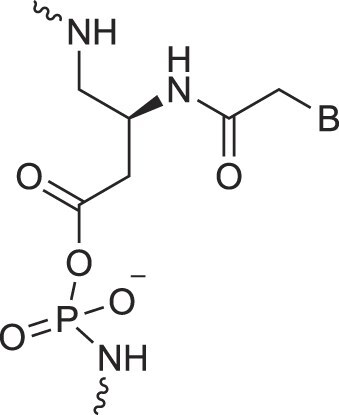	[140]
Prolinol nucleic acids	ProNA	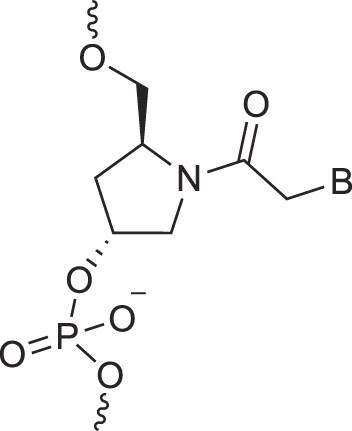	[141]
Q			
R			
Ribose nucleic acids	D-RNA (natural)	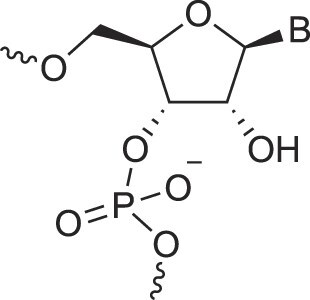	
	L-RNA (mirror image RNA)	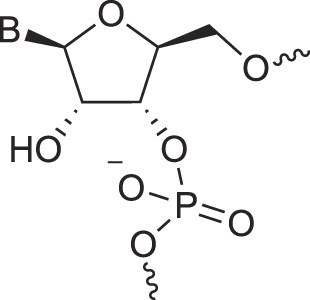	[142]
Ribulo nucleic acid	RibuloNA	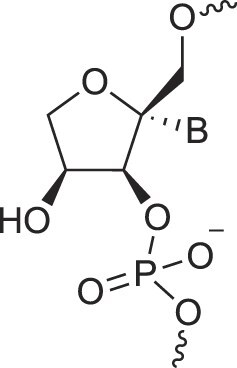	[143]
S			
Serinol nucleic acids	SNA	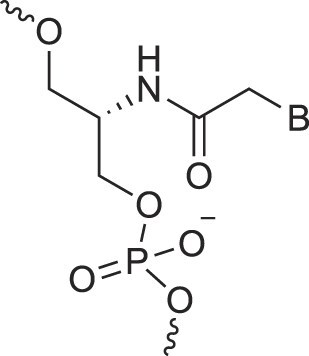	[144]
South methanocarba DNA	SMDNA	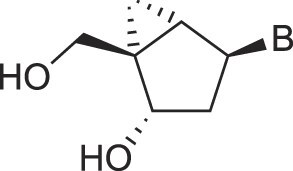	[145]
T			
*α*-L-threofuranosyl nucleic acids	TNA	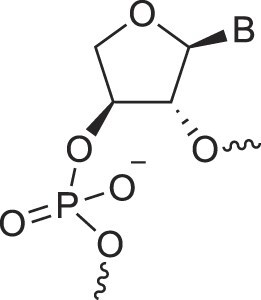	[29]
Threoninol nucleic acids	*a*-D-TNA	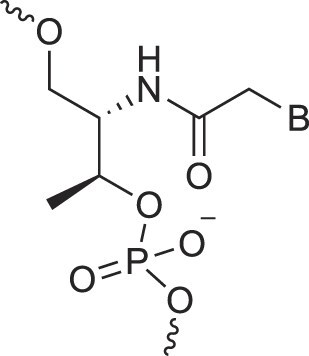	[146]
	*a*-L-TNA	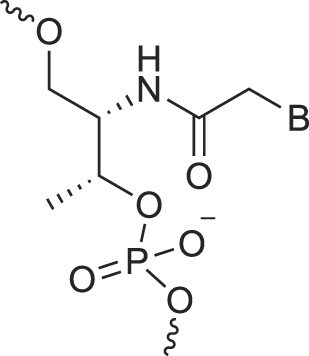	[146]
Tricyclo nucleic acids	TricycloDNA	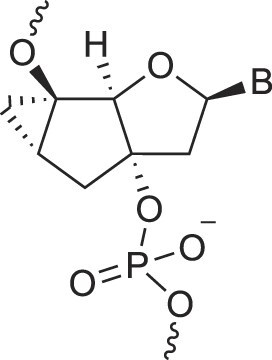	[147]
4′-Thio DeoxyNA	4′-ThioDNA	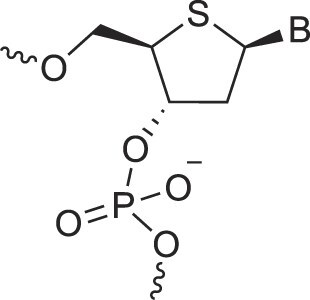	[148]
4′-Thio NA	4′-ThioRNA	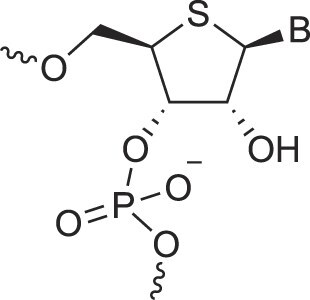	[149]
Triethyl amino NA	TEANA	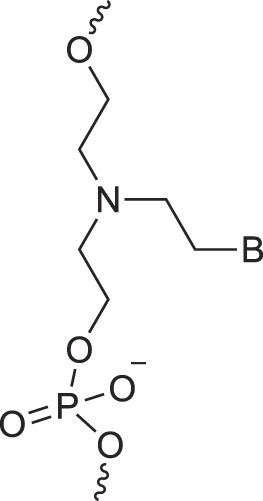	[99]
U			
Unlocked nucleic acids	UNA	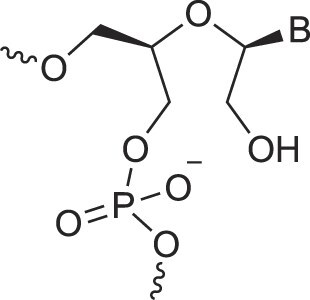	[150]
V			
Vinylpropyl nucleic acids	VPNA	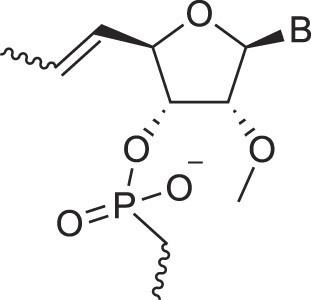	[151]
W			
W-shape nucleic acids	WNA	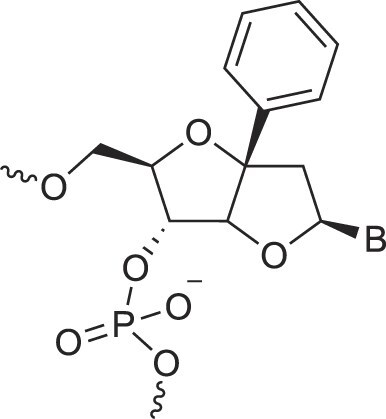	[152]
X			
Xylo nucleic acids	XyloNA	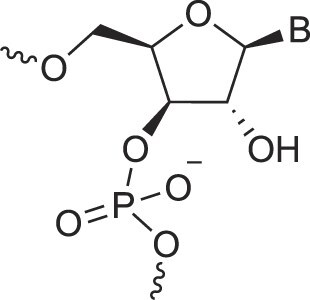	[31]
Deoxyxylo nucleic acids	dXyloNA	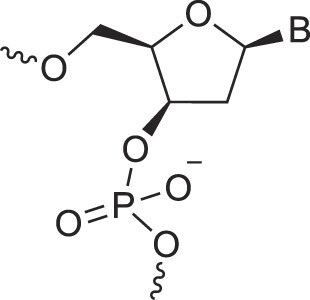	[153]
Y, Z			

N3'→P5' phosphoramidate DNA, where O3' is replaced by an amino group, bridges the properties of DNA and RNA. The presence of N3' weakens the gauche effect between O3' and O4', which is present in DNA (Fig. 4A), shifting the N-S pucker equilibrium to an RNA-like C3'-endo conformation [46]. The crystal structure of a fully modified phosphoramidate DNA dodecamer duplex confirmed that all amino sugars adopt the C3'-endo pucker. This structure also reveals an extensive hydration network around the backbone, facilitated by the 3′-NH group [47]. Additionally, amino groups interact with chloride anions, distinguishing the N3' hydrogen from its lone pair, which is positioned for maximum overlap with the antibonding P-O5' σ* orbital. This highlights the significance of the anomeric effect in DNA and RNA backbones and underscores the favorable stereoelectronics underlying the g-/g- conformation of α/ζ torsion angles around the P-O5' and O3'-P bonds (Figs. 1,4). Notably, phosphoramidate DNA not only emulates RNA conformationally but also functionally. For example, N3’→P5’ phosphoramidate DNA analogs of HIV-1 TAR and RRE RNA bind tightly and specifically to the RNA-binding Tat and Rev peptides, respectively [48].

Ribonucleotides exhibit significant flexibility, in part, based on the sugar pucker C3'-endo ↔ C2'-endo equilibrium. Beyond steric effects, their sugar conformation is influenced by a complex interplay of stereoelectronic effects, including the aforementioned four gauche effects, one anomeric effect, and the electron-withdrawing impact of the 2′-OH and 3′-OH groups [40]. The strength of the anomeric effect and the N-C1'-C2'-O2' gauche effect is also base dependent. This flexibility allows RNA sugar conformation to be influenced by internal physicochemical interactions and external factors, which may contribute to the catalytic power of RNA. Such interactions include 2′-OH lone pair interactions with vicinal phosphates [40], base stacking, H-bonding, hydration, electrostatic interactions, and steric effects [49]. Additionally, modifications in the electronic properties of nucleobases affect the strength of stereoelectronic effects, which, in turn, impact sugar and phosphate conformations [50, 51]. RNA can thus be envisioned as a molecular wire, transmitting stereoelectronic effects through a cascade of orbital overlaps involving bonding, non-bonding, and antibonding orbitals [52].

2′-5′-linked RNA has a strong cross-pairing preference for RNA over DNA [53]. That 2′-5′ RNA adjusts well to the structure of RNA extends to the functional realm. Thus, the concern that all RNA template-directed syntheses result in a complementary strand that contains a mixture of 2′-5′ and 3′-5′ linkages is lessened by the observation that functional RNAs tolerate a non-heritable 2′-5′ and 3′-5′ backbone heterogeneity [54]. The outcomes of MD simulations support a greater conformational flexibility of the furanose in 2′-5′ linked RNA, whereby the C2'-endo is preferred over the C3'-endo pucker [55]. This is fully consistent with crystal structures of RNA duplexes that contain several 2′-5′-linked residues, i.e. most of the these display the C2'-endo pucker, but a few adopt the C3'-endo pucker [56]. Conversely, all standard 3′-5′-linked riboses show the C3'-endo pucker that is the rule in standard A-form RNA.

**Figure 5.**
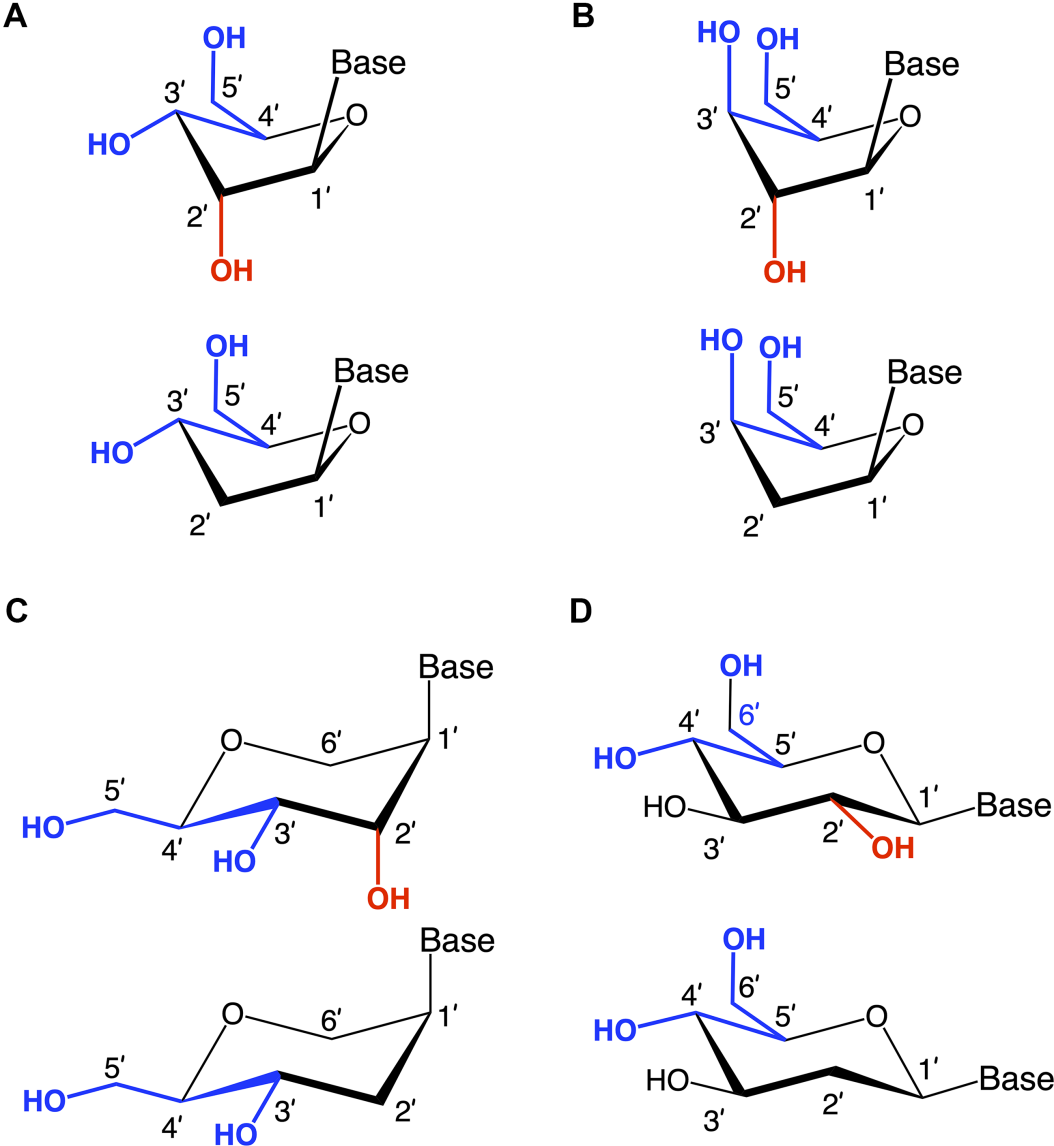
Nucleic acid pairing systems and their 2′-deoxy analogs. (**A**) RNA (top) and DNA (bottom), (**B**) XyloNA (top) and dXyloNA (bottom), (**C**) AltNA (top) and HNA (bottom), and (**D**) GlucoNA (top) and dGlucoNA (bottom). 2′-Hydroxyl groups are highlighted in red and backbone bonds are highlighted in blue (5′→3′, panels A-C, or 6′→4′, panel D).

RNA’s physicochemical uniqueness is reflected in its central biological role, particularly in catalysis. This is evident in its greater catalytic power compared to a six-membered XNA congener such as altritol nucleic acid (AltNA), as observed in intermolecular recombination experiments [57]. Whereas RNA demonstrates remarkable conformational flexibility, removing the 2′-hydroxyl group (as in DNA) results in a more rigid and well-ordered system. It is rare to find another sugar pair that achieves similar properties while retaining stable self-pairing and cross-pairing capabilities. Alternative sugar backbones, such as 6′→4′ glucopyranosyl nucleic acid, 2′,3′-dideoxyglucopyranosyl nucleic acid (homo-DNA), or hydroxy-hexitol nucleic acid (AltNA), fail to replicate RNA-DNA pairing properties. Among possible alternatives, the XyloNA/dXyloNA pair comes closest, as XyloNA is relatively rigid while dXyloNA is more flexible (Fig. 5) [31, 58].

Vicens and Kieft's argument that RNA G:U, G:A, and G:G pairs should not be considered mere mismatches is reasonable, given RNA’s ability to adopt an extensive range of folded structures [5]. These pairs not only fit into RNA’s folding landscape but actively enable its structural diversity. Furthermore, RNA rivals DNA in forming complex multistranded structures, including triplexes, quadruplexes, i-motifs, and Z-RNA [59]. The widespread presence of chemical modifications in RNA further directs its pairing modes [60], enhances stability and fidelity, and regulates the 2′-hydroxyl group's interactions with bases, base pairs, and higher-order structures.

Inside the cell, RNA exists in two major forms: messenger RNAs (mRNAs), which serve as templates for protein synthesis, and regulatory/catalytic RNAs. While mRNAs constitute only a small fraction of total RNA, ribosomal RNA (rRNA) represents the majority. Numerous non-coding RNAs have been identified, including rRNA, tRNA, miRNA, siRNA, lncRNA, circRNA, snRNA, gRNA, snoRNA, and tRFs [61]. The ligand-binding domain of riboswitches can be considered a natural RNA aptamer, and many of these RNAs are promising targets for drug discovery. Additionally, several RNA types undergo chemical modifications. For instance, 2′-*O*-methylation occurs in mRNA, tRNA, rRNA, and snRNA, contributing to RNA folding, structural stability, and function [62]. Such modifications, like 2′-*O*-methyl purine nucleosides, were incorporated into Macugen (pegaptanib), a 27-mer RNA aptamer functioning as a VEGF antagonist [63]. More recently, Izervay, which shares 2′-F, 2′-*O*Me and PEG modifications with Macugen, received approval against complement protein C5 for treatment of geographic atrophy in the eye [64]. These chemical modifications enhance aptamer stability against endonucleases and influence its three-dimensional structure, which remains an area of active investigation.

## Artificial genetic polymers

Inspired by nature, chemists have spent the last 50 years systematically designing and synthesizing a vast array of sugar-modified nucleic acids. The entries provided in Table 1 were identified from the literature as genetic systems that collectively form what we now describe as the XNA alphabet. This table is limited to XNA systems that have been incorporated into oligonucleotides. Most have been the subject of limited biochemical studies, which may include their influence on the thermal stability of a DNA or RNA duplex. Others have been synthesized as oligomeric XNA strands, and only in those cases has the full potential of the modification been revealed. Some of the more common examples of this series are FANA, TNA, HNA, ceNA, AltNA, tricyclicNA, LNA, homoDNA, pRNA, SNA, and aTNA, which have been evaluated in applications beyond simple recognition studies involving complementary Watson–Crick base pairing. We expect this table to grow overtime to include new examples, and we encourage members of the community to share their latest contributions with the co-authors of this review.

## Role of the sugar moiety in duplex formation

The sugar moiety of a nucleoside determines an oligonucleotide's conformation and hybridization properties. Various regio- and stereoisomers can arise in furanose nucleosides, and oligonucleotides derived from sugar-modified nucleosides exhibit distinct hybridization patterns. For example, in DNA, deoxyribose has a 1[*R*], 3[*S*], 4[*R*] configuration, enabling sequence-selective hybridization with both DNA and RNA with an antiparallel strand polarity. In contrast, α-DNA, with a 1[*S*], 3[*S*], 4[*R*] configuration, hybridizes sequence-selectively with DNA but in a parallel strand orientation [154]. DNA can also form parallel duplexes with itself [155], but RNA duplexes are strictly antiparallel, a difference that has its origin in their distinct backbone-base inclinations (Fig. 2). In dXylo nucleic acids, where the sugar adopts a 1[*R*], 3[*R*], 4[*R*] configuration, DNA hybridization does not occur, making dXyloNA a fully orthogonal nucleic acid system that hybridizes exclusively with itself [58]. Similarly, 2′-5′-linked DNA features a sugar moiety in a 1[*R*], 2[*R*], 4[*S*] configuration, allowing RNA hybridization in an antiparallel strand orientation but not with DNA [156]. Interestingly, 2′-5′-RNA was found to bind to complementary ssRNA but only bind weakly, or not at all, to ssDNA [53, 157]. These findings underscore the view that sugar modifications, rather than base or phosphate modifications, are key to designing orthogonal XNAs.

### Sugar pucker

**Figure 6.**
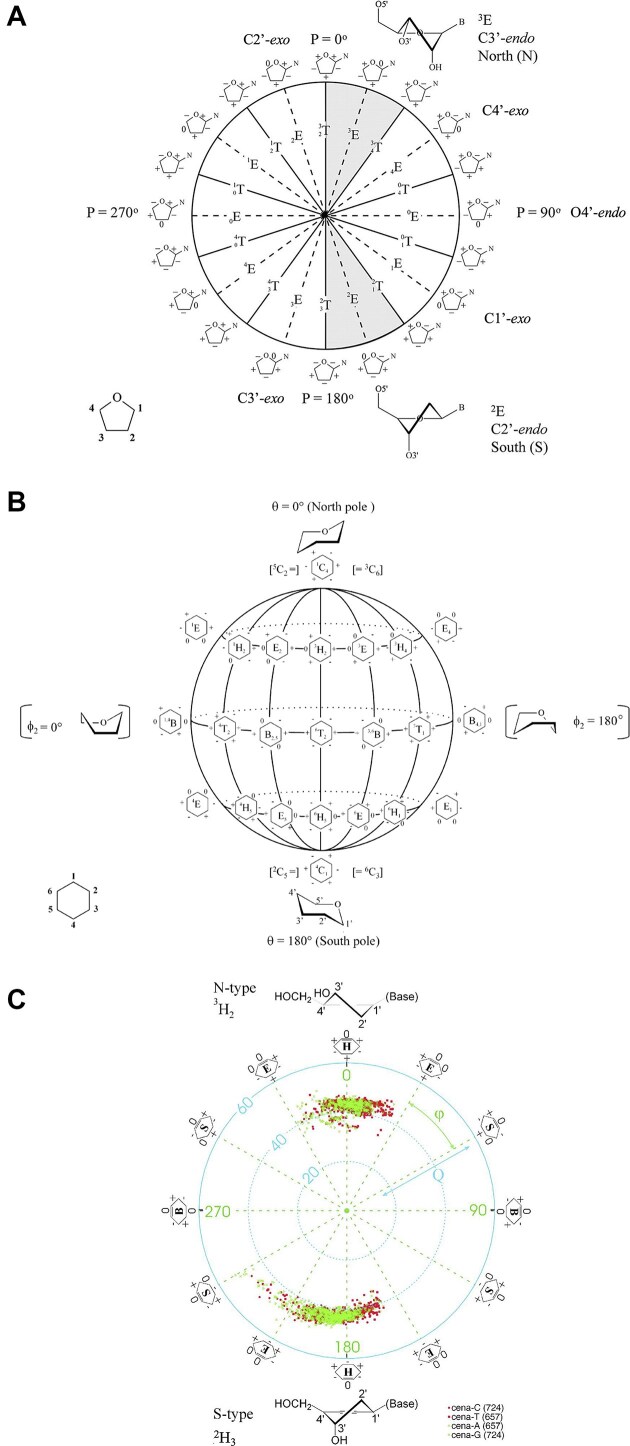
Conformational characterization of five-membered and six-membered (pyranose and cyclohexene) rings (**A**) Pseudorotation phase cycle of the furanose ring [173]. The preferred puckers of ribose and 2′-deoxyribose are shown at the top (**N**) and bottom (**S**), respectively. Points on the circle mark specific values of the pseudorotation angle P. Shaded regions represent conformations found in A‐ and B‐form helices. Riboses on the periphery of the cycle indicate signs of the endocyclic torsion angles ν_0_ to ν_4_: (+) positive, (–) negative and (0) 0°. (**B**) Pseudorotation globe of the cyclohexane ring [174]. The preferred conformations of pyranose are shown at the top (**N**) and at the bottom (**S**). Six‐membered ring conformations are shown on the front surface of the globe with signs of the endocyclic torsion angles ν_0_ to ν_6_ indicated: (+) positive, (–) negative and (0) 0°. (**C**) Pseudorotational phase cycle of a cyclohexene ring [174]. Data points for 2775 randomly generated and energy-minimized structures of C, T, A, and G nucleotides (see color code at bottom right) are shown and demonstrate that the energetically most favorable conformations of the cyclohexene ring are of the ^3^H_2_ (**N**) and ^2^H_3_ (**S**) types. Reproduced with permission from [175, 176].

This section provides an overview of the conformational properties of five-, six-, and seven-membered sugar rings. Sachse was the first to propose that the chair conformation eliminates strain in a planar cyclohexane ring [158]. A five-membered ring has two stable conformations—half-chair and envelope—each with ten possible forms, as represented in the pseudorotational wheel of a five-membered ring. Kilpatrick [159] coined the term “pseudorotation” to describe the hypothetical motion of an out-of-plane atom around the ring (Fig. 6A). This motion is characterized by two coordinates: the ring puckering amplitude and the pseudorotational phase angle (P) [159]. However, when a five-membered ring is substituted, as in modified nucleosides, pseudorotation becomes restricted—a phenomenon described by Altona and Sundaralingam [160]. According to their pseudorotational wheel model, the C3'-endo/C2'-exo twist conformation corresponds to *P* = 0° (type N), while the C2'-endo/C3'-exo twist corresponds to *P* = 180° (type S). Natural β-nucleosides primarily adopt *N*-type or *S*-type conformations in dynamic equilibrium. Most conformationally restricted nucleosides described in the literature favor the *N*-type conformation, as it enhances hybridization with RNA, which naturally adopts an *N*-type conformation in duplexes (Figs. 4, 6A). For example, TNA nucleosides adopt an *N*-type, C4'-exo conformation [29], explaining their preferential hybridization with RNA over DNA [41, 42]. In contrast, S-type sugar nucleoside analogs are rare, with examples including 3′-β-Me-cordycepin [161] and α-L-ribo-LNA nucleoside [128] (see Table 1 for structures). Others are AraNA (C1'-exo) [162] and α-LNA (C3'-exo) [128]. By contrast, the FANA analog displays an East O4'-endo sugar pucker (Fig. 6A) [24].

Cremer and Pople defined ring puckering coordinates for six-membered rings, which predominantly adopt chair conformations [163]. Chair-chair interconversion occurs via boat and twist conformations, the three fundamental forms of a six-membered ring, alongside half-chair and envelope conformations. The conformational landscape of a six-membered ring is described by three parameters: the degree of puckering (q), the pseudorotational phase angle (P), and the total puckering amplitude (Q), with Θ representing the degree of distortion (Fig. 6B). The two poles of the conformational globe correspond to the two chair conformations. The North pole represents the sugar conformation of HNA [28], which hybridizes with DNA and RNA, mimicking a natural nucleoside in the N-type conformation. The South pole corresponds to the sugar conformation of homo-DNA, which does not hybridize with DNA or RNA, making it an example of an orthogonal nucleic acid [164, 165]. Intermediate conformations, such as the 4′,6′-methano and 1′,6′-methano carbocyclic nucleosides, have also been described [145, 166] (North and South methanocarba NA, respectively, Table 1). These adopt boat-like conformations near the equatorial plane of the conformational globe. The 4′,6′-methano nucleoside mimics a furanose nucleoside in its C2'-exo conformation, stabilizing DNA/RNA duplexes, while the 1′,6′-methano nucleoside mimics a furanose nucleoside in its C3'-exo conformation, destabilizing them. As observed in furanose-type nucleosides, pyranose-type nucleosides also exhibit hybridization properties dictated by sugar substitution and conformation, enabling orthogonality in both sugar types.

Cyclohexene rings present a unique case, as their two sp²-hybridized carbon atoms restrict conformational flexibility. Consequently, cyclohexene rings tend to be locked in energy minima along an elliptical trajectory of boat and half-chair forms, similar to five-membered rings. The ^2^H_3_ conformation of a cyclohexenyl nucleoside mimics a C2'-endo furanose nucleoside, while the ^3^H_2_ conformation resembles a C3’-endo furanose nucleoside, with a low interconversion energy barrier [167] (Fig. 6C). The ^3^H_2_ conformation is stabilized by a π−σ* interaction, motivating the synthesis of cyclohexenyl nucleic acids (CeNA, Table 1), which hybridize with both DNA and RNA [87]. X-ray structures of CeNA/DNA hybrids reveal the coexistence of both half-chair conformations [168].

Oligonucleotides with 7′-5′ linked seven-membered sugar rings, such as oxacycloheptane (oxepane, OxNA, Table 1), have also been synthesized and tested for heteroduplex formation with DNA (oT15:dA15) and RNA (oT15:rA15), as well as their ability to elicit RNase H cleavage of RNA strands in hybrids [138]. The seven-membered ring is expected to be more flexible than five- or six-membered rings, adopting conformations such as chair, twist-chair, boat, twist-boat, and intermediate forms [169]. Theoretically, twist-chair conformers are energetically favored due to minimal steric interactions [170]. A crystal structure of oxepane confirmed its twist-chair conformation [171]. However, applying Cremer and Pople's four ring-puckering parameters (q2, q3, Φ2, and Φ3) [50] to the oxepane crystal structure showed that its conformation is best described as an intermediate between twist-chair and twist-boat [171]. Subsequent studies of oligonucleotides containing oxepane-thymidine revealed significant differences in pairing behavior and stability depending on backbone connectivity, including 7′-5′ (OxT0), 7′-5′ with 4′ and 3′ hydroxy groups (OxT1), 7′-4′ with 5′ and 3′ hydroxy groups (OxT2), and 7′-3′ with 5′ and 4′ hydroxy groups (OxT3) [172]. Molecular dynamics simulations indicated that each oxepane variant exhibits distinct conformational preferences, with varying degrees of twist-chair occurrence.

### Backbone inclination angle

Nucleic acid structural parameters can be categorized into those that define the local geometry of base pairs and those that impact the geometry of the helix [177]. The latter are particularly crucial for assessing structural orthogonality and the potential for self- and cross-pairing of XNAs. Among these, the inclination angle (*η*) quantifies the relative orientation of the base pairs with respect to the global helical axis. The backbone-base inclination angle (*η*_B_) is specifically defined as the relative orientation between the normal of the base or base pair and the BSpline backbone curve, which is traced by the phosphorus atoms of an oligonucleotide strand as it intersects the base plane [178] (Fig. 2).

**Figure 7.**
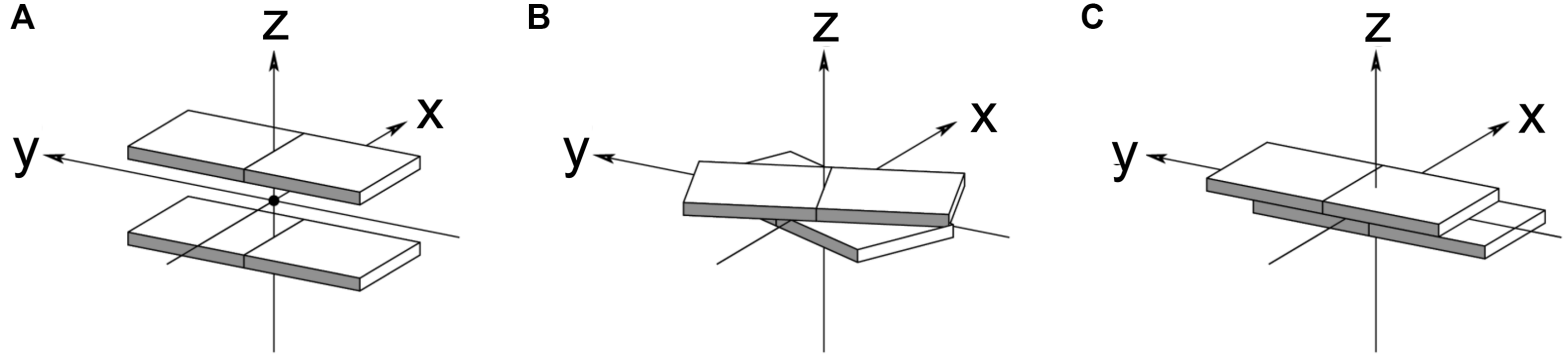
Definition of nucleic acid helical structural parameters (**A**) rise, (**B**) twist, and (**C**) slide [2]. Illustrations adapted from [3, 179].

**Figure 8.**
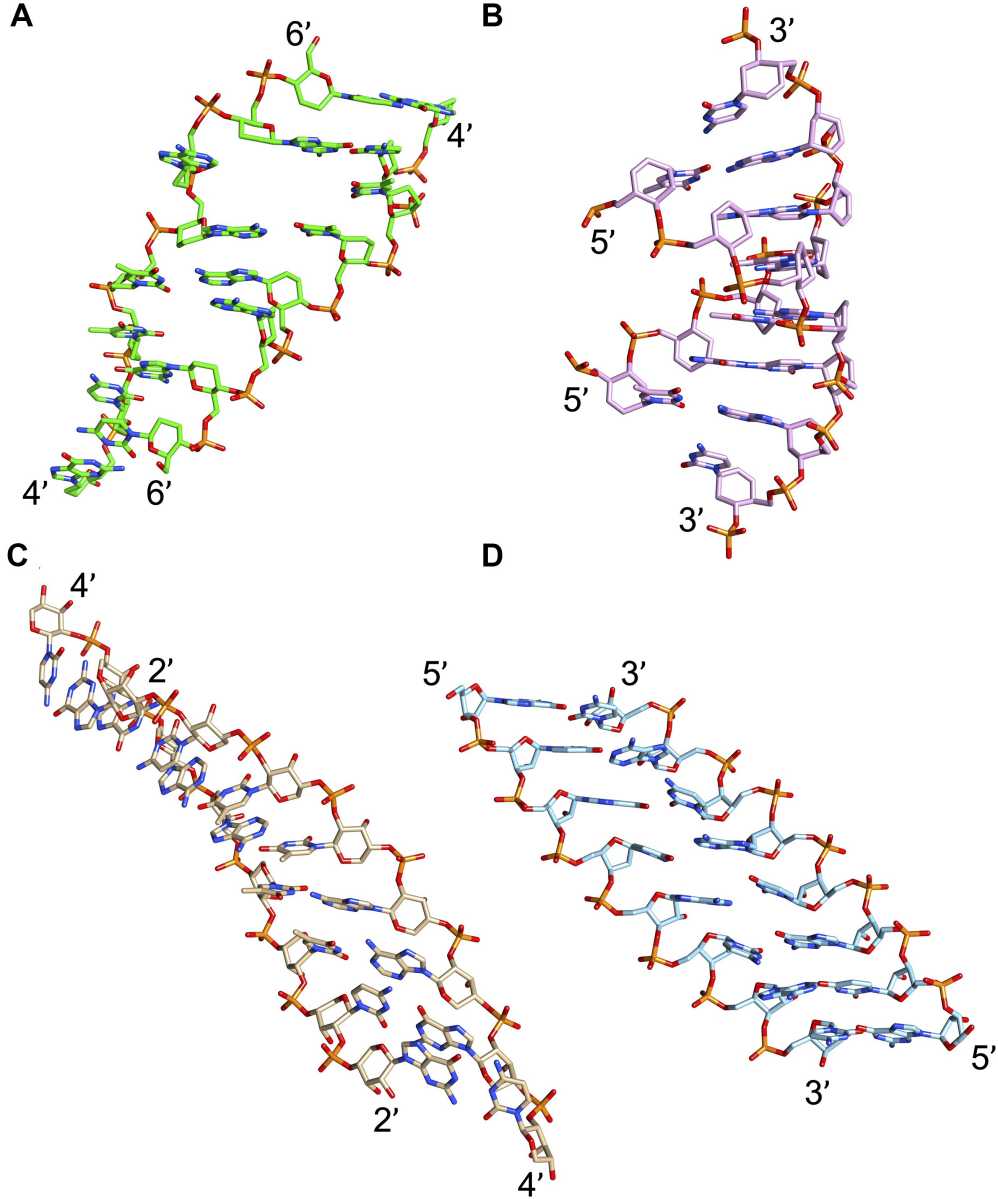
Structural models of orthogonal XNA pairing systems that display low helical twist and concomitant enhanced slide and interstrand stacking (homo-DNA, pRNA, XyloNA) or are left-handed (L-CeNA). (**A**) homo-DNA 6′-dd(CGAATTCG)-4′, X-ray structure, PDB ID 2H9S [164]. (**B**) L-CeNA 5′-c(GTGTACAC)-3′, X-ray structure, PDB ID 2H0N [180]. (**C**) pRNA 4′-p(CGAATTCG)-2′, NMR solution structure [181]. (**D**) XyloNA 5′-x(GUGUACAC)-3′, NMR solution structure, PDB ID 2N4J [31].

While B-form DNA inherently lacks backbone-base inclination, A-form duplexes (both DNA and RNA) exhibit inclination angles exceeding −30° (Fig. 2). The extent of inclination in XNAs is particularly evident in low-twist pairing systems (Fig. 7), such as homo-DNA (*η*_B_ = +35°) [15, 164] and pRNA (*η*_B_ = −46°) [30]. Increasing backbone-base inclination in XNAs enhances selective hybridization—favoring self-pairing over cross-pairing with DNA/RNA—and contributes to duplex stability through interstrand stacking, acting as a form of molecular glue (Fig. 8). Similarly, reducing helical twist (which in turn increases base pair rise) promotes selective hybridization and structural orthogonality (Fig. 7). The helical twist angle is primarily determined by the δ angle of the sugar moiety (e.g. ribose or 2′-deoxyribose, Figs. 1 and 4) and the phosphate geometry. The feasibility of achieving orthogonality depends on the intricate interplay between inclination and twist parameters and their capacity to establish an XNA geometry that is inherently distinct from dsDNA and dsRNA (Fig. 8).

## Duplex geometries

### Self-pairing versus cross-pairing

**Figure 9.**
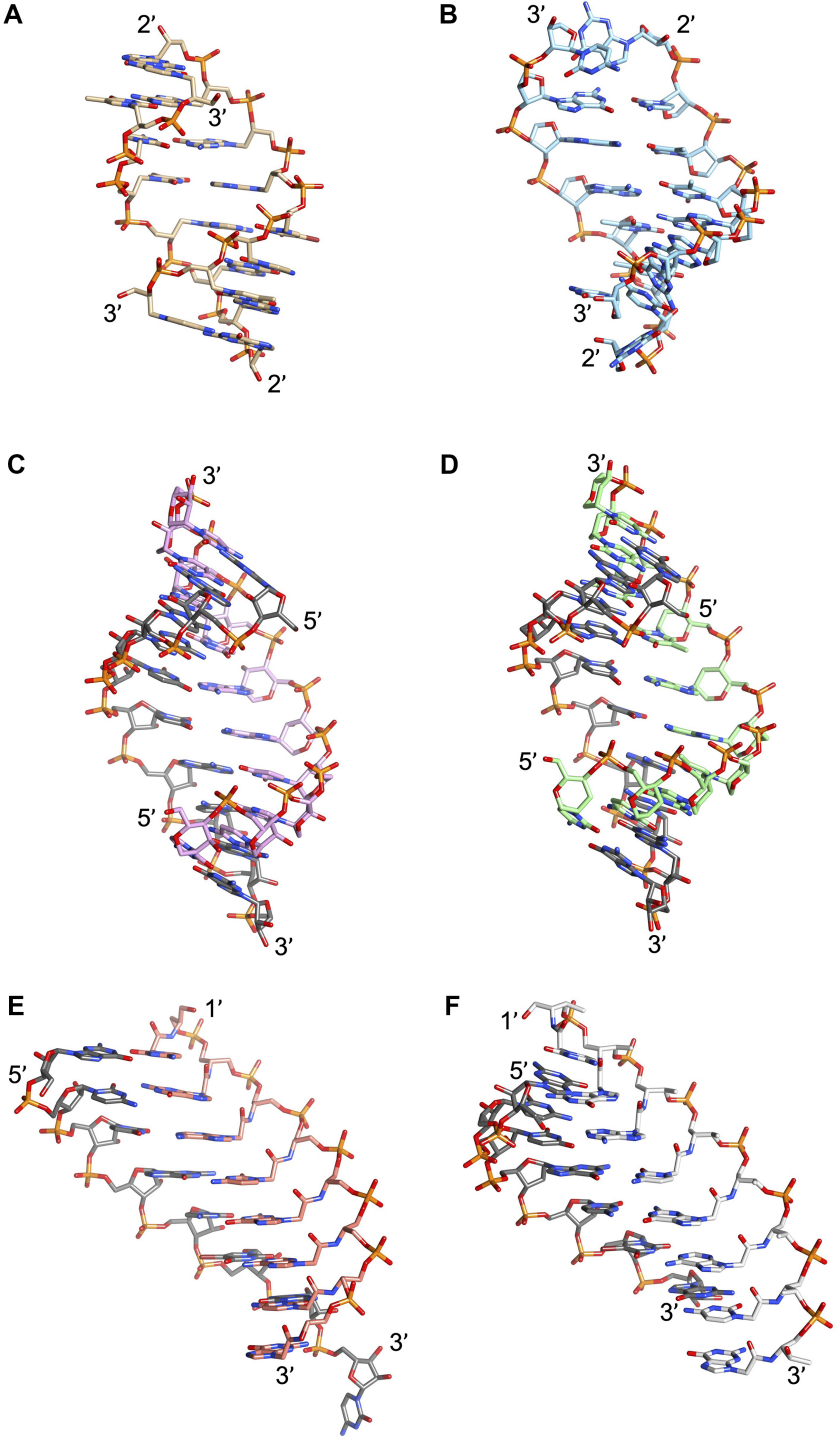
Structures of XNAs that cross-pair with RNA. (**A) (S**)-dsGNA 3′-g(CTC(Br)UAGAG)-2′, X-ray structure, PDB ID 2XC6 [187]; note that the C5-methyl group of T points into the minor groove as (**S**)-GNA adopts a right-handed duplex conformation with an inverted orientation of base pairs like in Z-DNA. (**B**) dsTNA 3′-t(CGAATTCG)-2′, NMR solution structure [43]. (**C**) AltNA:RNA 5′-alt(CCGUAAUGCC-P)-3′ : 5′-r(GGCAUUACGG)-3′, X-ray structure, PDB ID 3OK2 [185]. (**D**) HNA:RNA 5′-h(CCGTAATGCC)-3′ : 5′-r(GGCAUUACGG)-3′, X-ray structure, PDB ID 2BJ6 [183]. (**E**) SNA:RNA 3′-s(GCAGCAGC)-1′ : 5′-r(GCUGC(Br)UGC)-3′, X-ray structure, PDB ID 7BPG [192]. (**F**) aTNA:RNA 3′-at(GCAGCAGC)-1′ : 5′-r(GCUGC(Br)UGC)-3′, X-ray structure, PDB ID 7BPF [192]. Carbon atoms of RNA strands are colored in gray.

Most XNAs described to date are self-pairing systems, forming antiparallel duplexes that are governed by standard Watson–Crick base pairing rules. Cross-pairing between different XNAs or between XNAs and RNA or DNA only occurs when their helical geometries share similar inclination angles and twist parameters. Table 2 presents these parameters for XNAs that cross-pair with RNA, derived from X-ray structures or, when unavailable, NMR studies. Notably, the twist and inclination values for these dsXNAs closely resemble those of dsDNA and dsRNA (e.g. (*S*)-GNA and TNA Fig. 9A,B), with the exception of dsHNA (structure 2), which exhibits reduced helical twist, minimal inclination, and a slightly unwound duplex (15 base pairs per turn). This demonstrates that even conformationally restricted XNAs retain some structural malleability.

**Table 2.** Helical twist and inclination angles for selected XNAs that cross-pair with RNA

XNA Reference	dsHNA 1 [182]	dsHNA 2 [164]	HNA:RNA* [183]	CeNA:RNA [184]	AltNA:RNA [185]	dsTNA [43]	P^N^ DN [47]
Method	X-ray	X-ray	X-ray	X-ray	X-ray	NMR	X-ray
Twist [°]	33.2	24.2	31.5	31.3	30	27.4	33.2
Inclination [°]	24.3	3.4	14	17.1	13.7	24.1	14.7

*Average of four different structures

A second class of XNAs includes those with low twist values, such as dsPNA (∼19° twist, yielding 18 base pairs per turn, a configuration unattainable in DNA or RNA) or large inclination angles, as seen in dsGNA (Table 3). Despite such deviations, these XNAs can still cross-pair with RNA due to their acyclic, flexible backbones, as demonstrated in dsGNA structures [186] (Fig. 9A). Interestingly, dsGNA exhibits negative inclination similar to RNA and the (*S*)-isomer of GNA hybridizes with RNA, but is unable to pair with DNA (no inclination, Fig. 2A) or homo-DNA that exhibits a strongly positive inclination (Fig. 8A). The structural differences between dsPNA and PNA:RNA hybrids further highlight the adaptability of PNA.

**Table 3.** Helical twist and inclination angles for selected XNAs with low twist and/or large inclination that still cross-pair with RNA

XNA Reference	dsGNA [187]	dsPNA [188]	PNA:RNA [189]
Method	X-ray	X-ray	X-ray
Twist [°]	22.9	19	26
Inclination [°]	-46	3	14

Table 4 offers examples of orthogonal XNAs that do not hybridize with RNA or DNA. From these data, it is evident that significant deviations in either twist or inclination preclude cross-pairing. Another key determinant is backbone flexibility: XNAs with non-acyclic sugar moieties exhibit limited rotation around one or more backbone angles, affecting their ability to hybridize. For instance, pentopyranose (4′-2′) oligonucleotides possess two fixed backbone angles, while pentofuranose (5′-3′) nucleic acids retain one backbone angle with restricted rotation, making the latter more conformationally adaptable.

**Table 4.** Helical twist and inclination angles of selected XNAs that do not cross-pair with DNA/RNA

XNA Reference	ds-L-RNA [190]	ds-L-CeNA [180]	ds-homo-DNA [164]	ds-apNA [191]	ds-pRNA [181]	ds-dXyloNA# [58]	ds-XyloNA [31]
Method	X-ray	X-ray	X-ray	NMR	NMR	NMR	NMR
Twist [°]	-31.0	-29.6	14.3	small*	19	3.1	10.7
Inclination [°]	14.3	12.7	44.2 (*η*_B_)	-50	∼ -40	-50.6	-45.2

*No significant helicity, although NMR experimental restraints do not define this parameter directly due to their short-range nature (coordinates not available). #Average value of two different structures.

The significance of inclination in hybridization was experimentally demonstrated by Eschenmoser and collaborators. The pentopyranosyl (4′-2′) oligonucleotide family exemplifies strongly self-pairing systems that cross-pair within their class but not with RNA [193]. Cross-pairing occurs only between nucleic acids with similar geometries. For example, pentopyranose nucleic acids (β-D-ribo, α-L-arabino, β-D-xylo, and α-L-lyxo) are predicted to adopt quasi-linear double strands with antiparallel strand orientation, comparable twist, and inclination [84]. However, despite their structural similarity, they are unlikely to cross-pair in an antiparallel duplex with homo-DNA due to the opposite sign of backbone inclination [4, 193, 194] (Fig. 8).

### Orthogonal nucleic acid pairing systems

Synthetic biology aims to design and create new living systems with useful applications in medicine, agriculture, and material sciences. To ensure that these synthetic systems do not interfere with natural ecosystems, their genetic information should be encoded in artificial nucleic acids that cannot communicate with DNA and RNA [195]. A coding strategy of this type would prevent unintended genetic exchange with the natural genetic information of the cell. Additionally, polymerases (Pols) used to synthesize orthogonal XNAs must not interfere with the natural replication of DNA and RNA. By using orthogonal XNAs, synthetic biology can achieve a higher level of safety [196].

In this context, orthogonality refers to the inability of artificial nucleic acids to exchange genetic information with DNA or RNA, meaning they can only pair with themselves. DNA and RNA adopt specific helical conformations, primarily A- and B-form structures. Modifying the sugar component of a nucleic acid can produce XNAs with duplex geometries that fall outside the conformational space occupied by natural nucleic acids (Fig. 8).

The key structural parameters that determine orthogonality are inclination (slide) and twist [197] (Fig. 7). These factors influence rise, which affects the stacking distance between nucleobases. For example, D-DNA and L-DNA are mutually orthogonal [198] because they have the same inclination but opposite twist—D-DNA is right-handed, while L-DNA is left handed. However, as seen with dsGNA, XNAs can exhibit structural flexibility, meaning that simply analyzing the geometry of a self-complementary duplex is not enough to determine orthogonality [187].

To confirm whether an XNA is orthogonal to natural nucleic acids, hybridization studies should be performed using XNA oligonucleotides with all four nucleobases (mixed sequences). Relying only on polyA:polyT hybrids can lead to incorrect conclusions, as demonstrated with L-DNA [198].

### Helical versus linear

**Figure 10.**
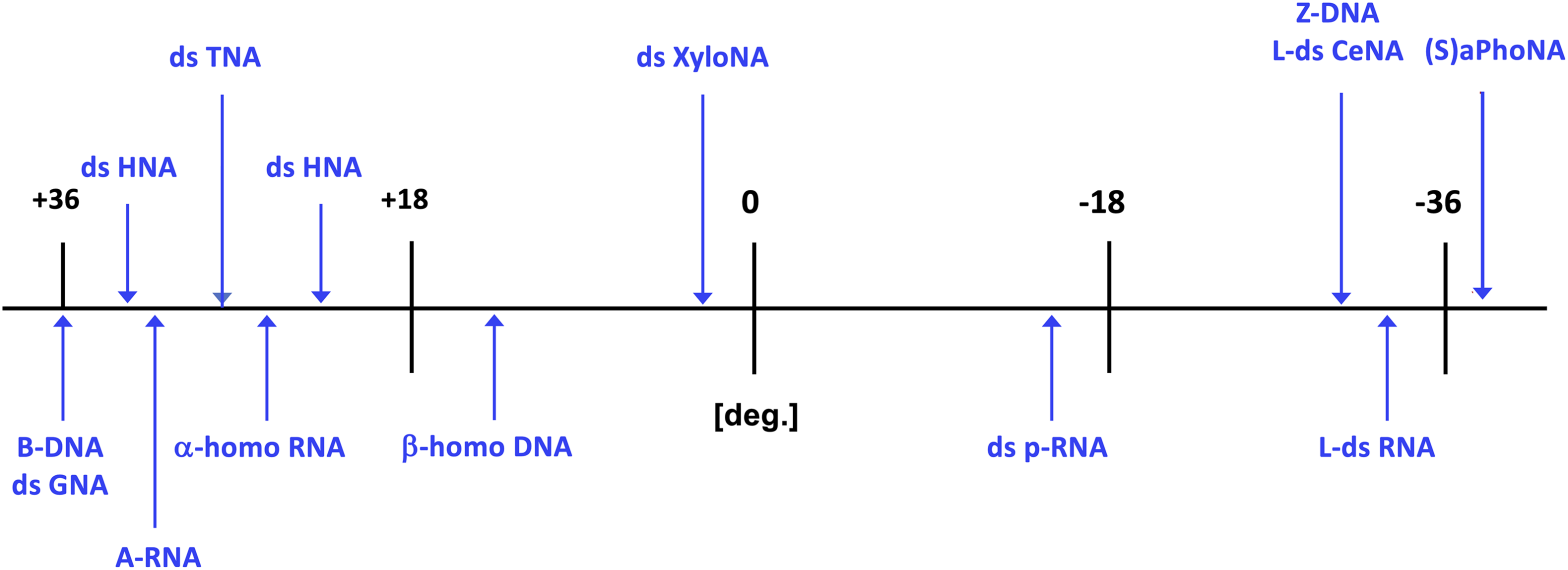
Average helical twist values (rotation/base pair in degrees) for DNA/RNA and selected XNAs.

The twist angle (Ω) is the parameter that determines the helicity of a double-stranded helix, while the repeat refers to the number of base pairs per helical turn. The twist angle represents the rotation of base pairs around the helical axis and can be positive (right-handed) or negative (left-handed) (Fig. 7B). However, it is not uniform along the helix—it varies depending on the nucleotide sequence [199]. This issue is less important in the context of the discussion about orthogonality, and we will use average values to describe the helicity of a double stranded helix. For example, a helical structure with 10 base pairs per turn and a rise of 3.4 Å (resulting in a pitch of 34 Å) will become a fully linear structure with an infinite number of base pairs per turn if completely unwound. As the helix unwinds, the base pair rise (Fig. 7A) increases. Because of this, nucleic acids with significantly different twist angles cannot hybridize with each other. Fig. 10 illustrates XNAs with twist angles ranging from +36° to −36° [175, 198] (see Table 1 for structures).

The dsXNAs provided in Table 2 reside on the left (West) side of the *x*-axis, indicating they cross-pair with DNA and/or RNA. One exception could be the α-homo-DNA:RNA duplex, which has a twist angle of 26.2° and an inclination of −1.78 Å. However, similar to α-DNA, it hybridizes with RNA in a parallel orientation, which complicates replication [197]. Aside from L-nucleic acids, promising candidates for an orthogonal genetic system include aPhoNA (dsZNA) [65], pRNA [181],and dXylo nucleic acids [58], which reside in the center or right (East) side of Fig. 10. Studies have shown that some Pols—such as Klenow exo- and Pol β SP20 can recognize modified nucleoside triphosphates as substrates, though only to a limited extent. Additionally, a trimer (one codon) has been successfully transliterated into DNA in *E. coli* [200]. However, to fully synthesize an XNA gene *in vivo*, it will be necessary to evolve mesophilic Pols capable of enzymatic XNA synthesis. So far, the complete chemical synthesis and expression of a fully modified gene in *E. coli* has only been demonstrated for base-modified oligonucleotides [201]. This finding raises new questions about the structural flexibility of dsXNAs, the role of hydration in duplex stabilization, and the polymerization process [202]. Because some flexibility is needed for Pols to evolve and support XNA synthesis, dXyloNA (Fig. 5) may be a better candidate for an artificial genetic system than pRNA (which is too rigid, Fig. 8) or ZNA (which is too flexible) [203].

### Role of chirality

The twist angle (and thus the helicity) is also an important factor determining hybridization between oligonucleotides of opposite chirality. For example, β-D-homo-DNA (polyA), with three stereogenic centers per nucleotide, forms heterochiral duplexes with β-L-homo-DNA (polyT) whereby the duplex is more stable than the homochiral system [204]. This heterochiral duplex displays virtually no twist and a strong backbone inclination as demonstrated using molecular modeling. β-D-Homo-DNA (polyA) also forms very stable duplexes with L-HNA (polyT) [114], most probably because HNA can also adopt a ladder-like conformation with equatorial orientation of bases and thus a duplex with low twist values [205]. Conversely, single-stranded GNA, with only one stereogenic center per nucleotide, is preorganized into a helical structure [206]. Like DNA and RNA strands of opposite chirality, (*S*)-GNA and (*R*)-GNA do not base pair with each other [110], because the single stranded oligomers feature an opposite helical twist. In this case, a mixed A/T sequence was studied. Thus, exchange of information between two homochiral oligomers of opposite chirality is a matter of their geometry, rather than of the type or numbers of their stereogenic centers.

PNA is achiral and can hybridize with both L-DNA and D-DNA [207]. Consequently, PNA can serve as a template for nonenzymatic L-RNA synthesis, representing an interesting case of informational transfer between two different XNAs [208]. SNA becomes chiral only upon incorporation into an oligonucleotide [209]. As a result, its chirality is sequence-dependent, with palindromic sequences remaining achiral. This also enables the same SNA sequence to hybridize with both D-DNA and L-DNA but in opposite orientations (5′→3′ and 3′→5′). However, enantiomeric oligonucleotide hybridization can become complex when the XNA exhibits extensive conformational diversity. For instance, D-cyclohexane nucleic acid hybridizes with D-DNA, whereas L-cyclohexane nucleic acid hybridizes with D-homoDNA, forming duplexes with distinct geometries [210].

## Biophysical properties

The stability of nucleic acid structures depends on various factors, such as sequence, H-bonding, stacking interactions, hydration, counter ions, preorganization of single-stranded (ss) oligonucleotides, and crowding conditions. Additionally, the thermodynamic behavior of nucleic acids is influenced by the out-of-equilibrium conditions that persist inside cells. These factors also affect the ability of nucleic acids to recognize Watson-Crick base pairs or mismatched pairs like wobble or Hoogsteen base pairing modes. While these topics have been covered extensively in previous reviews, this section will focus on a few key examples from the XNA field (Table 1).

### Example 1: Conformational preorganization

XNAs with rigid backbone structures are more preorganized as single strands than XNAs with more flexible backbones, which gives them an advantage in forming stable double-stranded structures. Studies on HNA and AltNA compared to RNA and Mannitol Nucleic Acid (MNA) show that ssHNA and ssAltNA are preorganized into A-type structures (Fig. 9C and D), which are more stable than ssRNA [133]. This preorganization helps form more stable duplexes, including self-complementary XNA and RNA:XNA hybrids. MNA, on the other hand, adopts a conformation different from A- and B-forms, making it less effective at hybridizing with RNA [211]. Frequent H-bonds between the 3′-hydroxyl and the 6′-O of the phosphate backbone of the following base changed the conformation of the single strand as well as the MNA:RNA complex. The MNA:RNA backbone widens up and shows partial unwinding and disruption of base pair H-bonds consistent with their low hybridization potential. To achieve a higher state of preorganization, the sugar moiety of XNAs can be designed with bicyclic [20] or tricyclic sugar [212] structures or the ribose sugar can be modified to favor a C3'-endo conformation. This can involve substituting electronegative groups, like fluorine in the 2′-position or less electronegative groups like NH in the 3′ position [44].

Intrastrand stacking contributes significantly to nucleic acid stability by further organizing the backbone in a single stranded state. This stacking stabilizes duplexes that display C3'-endo sugar puckering. XNAs such as XyloNA [31] and pRNA [4] benefit from this type of stabilization. Acyclic nucleic acids generally do not hybridize well with DNA or RNA [209]. One exception is (*S*)-GNA, which can hybridize with RNA under certain conditions [213, 214]. Similar exceptions include PNA [25] and SNA [209] (Fig. 9E). PNA is especially notable for its ability to pair with both D- and L-DNA and RNA [207]. (*S*)-ZNA, another acyclic nucleic acid, can form stable self-complementary duplexes but hybridizes poorly with DNA. This behavior is partly due to the compactness of the ZNA structure. The reduced hybridization ability of (*S*)-GNA with DNA is attributed to the inverted orientation of GNA bases, which reduces H-bond formation with G:C pairs [214, 215].

Introducing chemical groups that restrict conformational flexibility, such as amido groups, can enhance hybridization between acyclic nucleic acids and DNA or RNA. For example, introducing a methyl group into the backbone of SNA produces threoninol nucleic acid (aTNA) [216] (Fig. 9F). This chemical modification increased duplex stability with DNA and RNA, especially for the L-isomer (L-aTNA), while the D-isomer (D-aTNA) is orthogonal to DNA and RNA [192, 216].

The preorganization of nucleic acids is also influenced by the phosphate in the backbone. The presence of certain conformations, such as gauche-trans and trans-trans orientations, impact free energy [217]. Introducing methylene phosphonate groups into the backbone increases flexibility [218] and changes sugar puckering [219]. Preorganization can also affect the susceptibility of nucleic acids to degradation by nucleases. Phosphodiesterases cleave a P-O bond in nucleic acids by an addition-elimination mechanism via the formation of a penta-covalent intermediate that generally collapses into 5′-phosphorylated fragments. The nucleophiles involved in this reaction can be diverse and range from water to protein side chains like Tyr, Ser, and His [220]. Nucleases can be specific for DNA or RNA or be nonspecific [221]. Nothing is known about the substrate specificity of nonspecific nucleases, and it is unclear whether such enzymes are capable of also degrading certain XNAs. Examples of sugar non-specific endonucleases are staphylococcal nuclease from *S. aureus* [222] and rat liver nuclease [223]. Most DNases are metal ion dependent nucleases and cleave nucleic acids in the base-stacked helical conformation [220]. Conformational preorganization of the single strand may help in binding of the nucleic acids to the enzyme and their absence may contribute to the enzymatic stability of acyclic nucleic acids. It is generally accepted that the most relevant nucleases in the context of therapeutic oligonucleotides are 3′-exonucleases, and as such, phosphorothioate [214, 224], 3′-terminal XNAs [225], and inverted 3′-3′ linkages [226] can stabilize the oligonucleotide against nucleolytic degradation.

### Example 2: Mismatch discrimination

Exchanging the 2′-deoxyribose sugar in DNA for a 2′,3′-dideoxyglucose sugar, such as the one found in homo-DNA results in altered base pairing priorities despite both systems having standard A, C, G and T nucleobases in both systems: G:C > A:T (DNA) versus G:C > A:A ≈ G:G > A:T > A:C (homo-DNA) [227]. The purine–purine (Pu:Pu) pairs in homo-DNA are of the reverse-Hoogsteen type. Thus, if one generated a very long homo-DNA of random sequence and the molecule adopted a folded state, it is very likely that there would be many reverse-Hoogsteen Pu–Pu pairs in addition to standard G:C and A:T pairs. In fact, it was found in the crystal structure of the homo-DNA octamer dd(CGAATTCG) [164] that one of the As is looped out of the duplex (Fig. 8A) and forms a reverse-Hoogsteen pair after it is inserted into the adjacent duplex opposite an orphaned T [178]. The other A forms an intermolecular reverse-Hoogsteen pair with T from the adjacent duplex. In the crystal the homo-DNA octamer engages in a dimer of dimers and there are multiple G:G pairs in the lattice. Consequently, the term mismatch is relative and somewhat dependent on the chemistry of the nucleic acid backbone.

Natural genetic polymers, especially RNA, can adopt a diverse range of base pair orientations [5]. Whenever RNA is being encoded (transcription) or decoded (translation), or in telomerase (guide), the spliceosome, the signal recognition particle, etc., the molecular machines doing the job have to keep RNA in a more-or-less canonically paired duplex state. If RNA were to adopt a complex 3D fold it needs to be unwound and reigned in as the fold is choked full of non-Watson–Crick pairs. Hence there must have been evolutionary pressure to transition to DNA for storing the genetic information as RNA is much too promiscuous in terms of pairing and stacking whereas DNA is faithful and monotonous by comparison.

A biologically relevant mismatch base pair is the G:U wobble pair in RNA. It serves many RNA functions such as in tRNA aminoacylation and ribosomal activity, splicing and ribozyme activity [228]. However, at the level of DNA wobble base pairing is unwanted as it leads to mutagenesis. A G:U wobble pair leads to a local structural perturbation of dsRNA. The glycosidic bond angle at C1' of the ribose sugar of W-C base pairs is ∼55°. In a G:U wobble pair these values are ∼40° for G, and ∼70° for U [228]. This leads to undertwisting and overtwisting, respectively, of the helix at the wobble position [229]. Therefore, one would expect that in conformationally more rigid XNA structures, mismatch discrimination will be higher and the fidelity of base pairing increased as less conformational dynamics will be allowed. Although this was only studied in an 8-mer duplex (which may cause large differences in T_m_ values dependent on the localization of the mismatch base pair in the duplex and the neighboring base effects), the ΔT_m_ values for mismatch discrimination were higher in the HNA:RNA duplex relative to the RNA:RNA duplex [230]. The ΔT_m_ between match and mismatch base pairs in the RNA:HNA duplex varied between –12.8° (C:A mismatch) and –38.2° (G:A mismatch). In the RNA:RNA duplex, the values varied between –3.9° (G:U mismatch) and –27.9° (G:A mismatch). Thus, the G:U wobble pair in dsRNA leads to a ΔT_m_ of –3.9°. Although the data cannot be strictly compared because in the HNA sequence U was replaced by T, the ΔT_m_ of the G(RNA):T(HNA) mismatch was –12.9°. In the reversed pair (G in HNA and U in RNA) the difference in ΔT_m_ was much lower, i.e. –1° in favor of HNA:RNA.

These preliminary limited data point to the possibility that the use of conformationally restricted XNAs as genetic polymers might produce increased fidelity of base pairing. When considering applications in synthetic biology, an important question is how the XNA sugar will influence base pairing “in vivo.” The intracellular environment is a gel-like matrix [231] and different from the conditions that are used for DNA Pol-catalyzed nucleotide incorporation assays *in vitro*. Initial studies have been performed using crowding conditions “ex vivo” [232] and using an ‘in vivo’ model in *E. coli* [233] with isoC^Me^ and isoG bases on DNA and HNA templates. This means that mismatch recognition is evaluated and not matched recognition as it is expected that isoC preferentially base pairs with isoG; in the *in vivo* assay only pairing with A, C, T, and G can be evaluated. The *E. coli* DNA Pol I Klenow fragment was used as surrogate enzyme in the *in vitro* assays and the difference in recognition pattern is sometimes small. For example, under crowding conditions and using Klenow fragment, CTP and dTTP were incorporated in almost equal quantities opposite the isoG base on an HNA template [232]. In Table 5, only the nucleoside triphosphate most efficiently incorporated is mentioned, independent of the difference relative to other NTPs. The data demonstrate that using PEG200 as the crowding agent does not allow perfect mimicry of *in vivo* conditions and results will likely deviate from those “*in vivo*.” In the present case, the selectivity of *in vivo* recognition of isoC^Me^ and isoG on a DNA template is the same as on an HNA template.

**Table 5.** Recognition of isoC^Me^ and isoG bases on DNA and HNA templates in insertion assays under different conditions using Klenow Fragment (-) as Pol. The substrates tested are dCTP, dTTP, dGTP, and dATP. The table only lists the nucleotide triphosphate that is most efficiently incorporated opposite the modified base

Template	*In vitro* conditions	Crowding conditions	*In vivo* (*E. coli*)
isoC^Me^ in DNA	dATP	dATP	dGTP
isoG in DNA	dTTP	dTTP	dTTP
isoC^Me^ in HNA	dGTP	dGTP	dGTP
isoG in HNA	dCTP	dCTP	dTTP

### Example 3: Hydration

The role of water in stabilizing nucleic acid structures has been well documented [234]. High humidity favors the B-form and low humidity favors the A-form [235]. The major groove is more hydrated than the minor groove and the water structure entails primary and secondary water layers [236, 237]. Water molecules in the primary layer exhibit a high residence time and this layer behaves as quasi crystalline water [238]. This also means that Watson–Crick base-pairing is additionally stabilized by H-bonding due to hydration. A G:C base pair involves three Watson–Crick H-bonds and is further stabilized by 8 water molecules in the grooves. In an A:U base pair, there are two Watson–Crick H-bonds and five water molecules in the grooves [239]. Clearly, water is a factor that should be considered when base-modified nucleic acids are evaluated in synthetic biology. The introduction of 7-deazapurine bases, for example, leads to dehydration of the major groove, thereby triggering increased conformational plasticity of the modified nucleic acids [202], which results in a change of the fidelity of base pairing [240].

### Example 4: Charge repulsion

Electrostatic repulsion between phosphate groups in nucleic acids is minimized by counter ions, such as divalent ions, which are more effective than monovalent ions like Na^+^. This repulsion can also be reduced by using XNAs with a neutral backbone, such as PNA, which hybridizes with DNA and RNA independently of ionic strength. The neutral backbone of PNA makes it a strong probe for strand displacement and for forming stable triplexes [188], though it has limited cellular uptake [241], which restricts its use in synthetic biology applications. Nonetheless, PNA remains a valuable tool for diagnostic purposes [242].

## Synthesis, replication, and evolution

The events that led to the origin and evolution of life on Earth rely on several key biological processes that include the diversification, replication, and propagation of genetic information in actively dividing cells. Since evolution occurs at the cellular level, genes that increased the fecundity of a cell were retained by natural selection, while those with deleterious properties were lost to extinction. As cells continued to grow and divide, protein secondary and tertiary structures are thought to have emerged as genetic sequences recombined in various ways to produce new types of proteins, including those with novel folds and functions [243–246]. The key biochemical reaction for replication is phosphodiester bond formation. In this reaction, the growing strand is recognized as a primer that is extended in the 5′-3′ direction by sequentially adding 5′-nucleoside triphosphates to the terminal 3′-hydroxyl group as dictated by the sequence of the complementary template [247]. Recombination is a more complex process that involves the biochemical steps of transesterification, phosphodiester hydrolysis, and intermolecular ligation, which allow segments of DNA and RNA to be shuffled or removed, as is the case for RNA splicing [248].

Some RNA splicing events catalyzed by ribozymes can also be performed by DNAzymes. Early examples included DNAzymes that cleave and ligate RNA substrates at specific nucleotide positions [249, 250]. When first discovered, these results demonstrated that DNA molecules, like RNA before it [251], have the capacity to fold into shapes with catalytic activity. Growth in this area led to the discovery of second-generation DNAzymes that function under simulated physiological conditions and can achieve persistent allele-specific gene knockdown activity of endogenous mRNAs in cells [252–254]. This approach relies on the unique physicochemical properties of XNAs to increase catalytic activity and biostability of the DNA scaffold. Engineered Pols have extended the types of genetic polymers that are capable of enzymatic catalysis to include a small but growing set of XNAs, like TNA, HNA, and FANA, that can be evolved in the laboratory to cleave and ligate RNA substrates [252, 255–257]. Expanding these systems to include the evolution of XNAzymes that can act on XNA substrates is an important area of future growth.

Nucleic acid biochemists have long appreciated the importance of the 2′ hydroxyl group on the ribose sugar as a reaction moiety for RNA transesterification. For example, all RNA-cleaving nucleic acid enzymes follow the same metal-dependent reaction mechanism to produce an upstream cleavage product carrying a 2′,3′-cyclic monophosphate and a downstream strand with a 5′ hydroxyl group. This reaction involves nucleophilic attack of a 2′ oxyanion on the neighboring phosphodiester bond, which can only be achieved by the deprotonation of a vicinal diol, like the one found on RNA. This unique mechanistic feature implies that only XNAs with vicinal diols are capable of recombination using ribozymes to cut and ligate RNA segments together. This hypothesis proved correct for altritol nucleic acid (AltNA, Fig. 5C) in a nonenzymatic reaction format that identified the *cis-*diol configuration shared by RNA as a fundamental determinant of hydrolysis [57]. This finding suggests that XNAs with a *cis-*diol on their sugar moiety are important model systems for prebiotic chemistry and possible RNA progenitor candidates for the evolution of life.

Nonenzymatic polymerization and ligation reactions have long served as a model for RNA synthesis in the absence of complicated enzymatic machinery [258]. These reactions follow a mechanism in which the 3′-hydroxyl group attacks a 5′-activated monophosphate on the incoming mononucleotide, or the case of ligation, a 5′-activated oligonucleotide. Leaving groups used to achieve phosphodiester bond formation include 2-methylimidazole [259], 2-aminoimidazole [260], oxyazabenzotriazole [261], and proline [262]. The nonenzymatic transfer of genetic information from RNA templates to XNA products have been demonstrated for a number of systems, including PNA [263], p-RNA [264], TNA [265–267], and MNA [268]. Likewise, HNA [269], AtNA [270], GNA [271], and TNA [272] offer examples of XNA templates examined for RNA transcription. Nonenzymatic replication within the same XNA system has been widely studied for RNA [273], but such examples are limited for XNAs other than DNA and RNA. However, systems that have been studied in this context include pRNA [274, 275] and the acyclic threonine derivative L-aTNA [276], which allow for pRNA ligation on pRNA templates and L-aTNA ligation on L-aTNA templates, respectively.

Further, in the context of ligation, it is noteworthy that some XNAs such as HNA, TNA, and LNAs can be accessed by enzymatic ligation reactions [266, 277–279]. Moreover, other chemoenzymatic methods are currently being developed for the synthesis of XNA oligonucleotides [280–282].

RNA-dependent RNA Pol ribozymes evolved *in vitro* offer valuable tools for studying XNA synthesis in the absence of protein enzymes. One interesting example is an RNA-dependent RNA Pol ribozyme that exhibits varying levels of promiscuity toward XNAs, both as monomers and as templates [283], implying that ribozymes could have enabled evolutionary transitions between different types of early genetic systems. A second important example is a cross-chiral RNA Pol ribozyme capable of copying RNA of the opposite chiral handedness, such that L-RNA information is copied into D-RNA and vice versa [284]. This class of ribozymes overcomes the well-characterized problem of cross-chiral inhibition in which nonenzymatic polymerization reactions of D-RNA were inhibited by the presence of L-RNA monomers [285].

Aptamers—nucleic acid molecules that mimic antibodies by folding into structures with ligand binding sites that are complementary in size and shape to specific targets—hold significant potential as future diagnostic and therapeutic agents [286–288]. Although some aptamers exist naturally as the ligand binding domain of riboswitches [289], most are identified by *in vitro* selection approaches that mimic Darwinian evolution [290]. While structural insights into the ligand binding properties of DNA and RNA aptamers are well established [291], often relying on induced fit to optimize intramolecular interactions for cognate target binding, very little is known about the ability for XNA aptamers to fold and function. Modeling of an HNA aptamer bound to hen egg-white lysozyme features a hGhT-rich motif with non-Watson-Crick interactions [292], suggesting a diverse repertoire of structural conformations may be available to XNA aptamers. Advances in the development of base-modified TNA aptamers have established parallelizable paths for generating high affinity sequences from single-round screening approaches mirroring those of DNA-encoded small molecule libraries [293]. The ability to query diverse library chemotypes while avoiding the need for iterative rounds of selection offers a promising strategy for accelerating the discovery of therapeutic aptamers.

Natural Pols including those from thermophilic organisms [294, 295] and trans-lesion and repair Pols [296] are in some cases able to handle certain XNAs, albeit often with reduced efficiency of catalysis. Early work in this area showed that certain DNA polymerases could synthesize short segments of DNA on TNA templates [297], while other polymerases could synthesize limited amounts of TNA on DNA templates [298–301]. Similar results were also observed for FANA and HNA replication systems [302–305]. Bst DNA pol (A-family), an analog of E. coli DNA polymerase I, stands out as an interesting natural polymerase due to its ability to reverse transcribe diverse XNA templates into DNA [306]. Indeed, this enzyme is still used today to reverse transcribe TNA into DNA [306]. These early studies laid the foundation for subsequent polymerase engineering efforts that made it possible to replicate XNAs in the laboratory by copying genetic information back and forth between DNA and XNA. For an exhaustive review on Pol engineering see reference [307].

**Figure 11.**
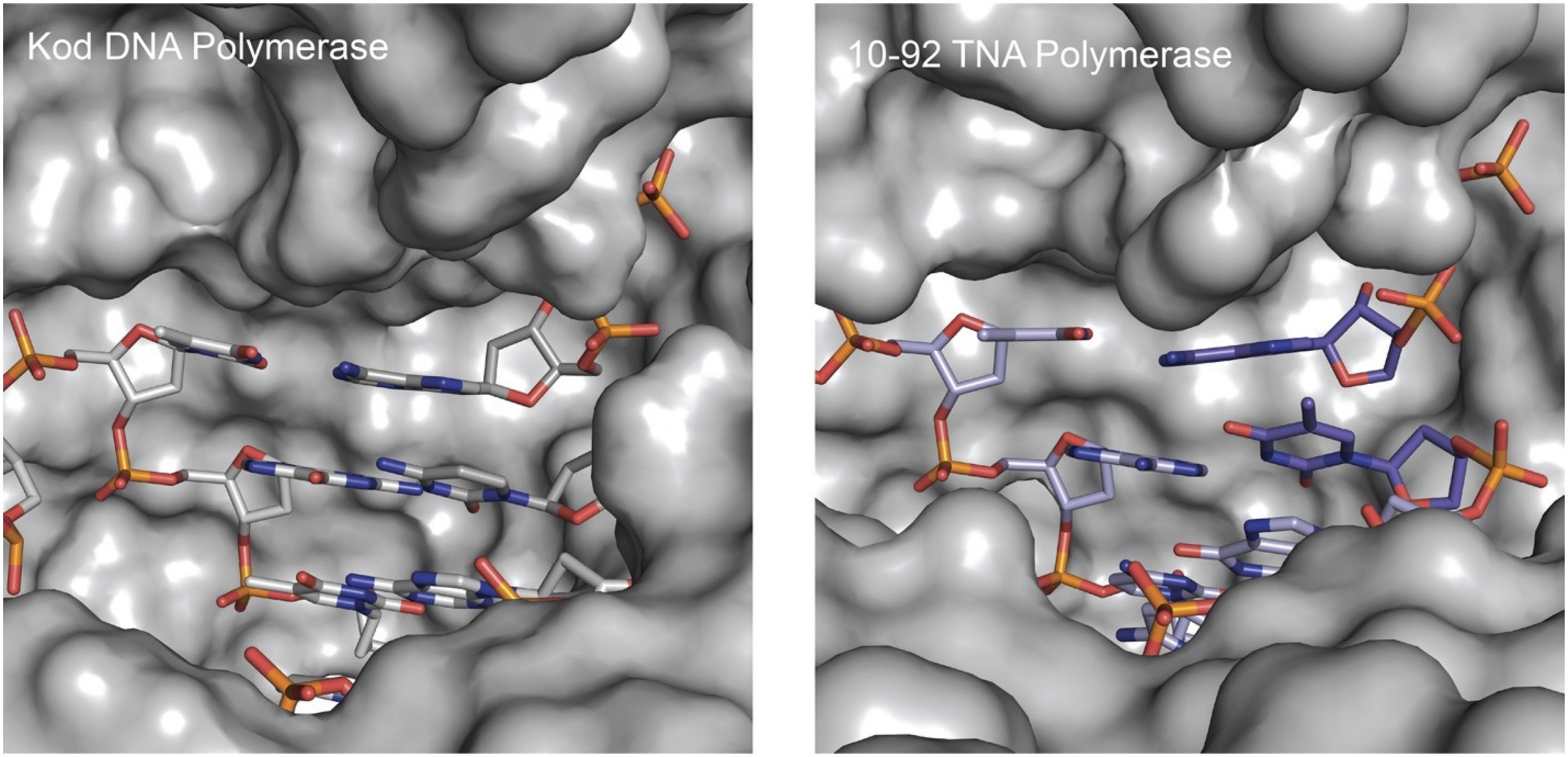
Active site view of natural Kod DNA Pol and the engineered 10–92 TNA Pol.

The development of engineered XNA Pols led to the isolation of TNA and HNA aptamers with specific protein-binding activity from large combinatorial libraries [308, 309]. Although an important landmark in the newly minted field of synthetic genetics [11, 310], the modest activity of first-generation XNA Pols limited their use in downstream applications [9]. XNA Pol reactions performed at that time required long extension times and the presence of manganese ions in the reaction mixture to reduce the substrate specificity of the enzyme against XNA, either in the template or as triphosphates [297, 298, 305]. Fortunately, continued advances in the development of Pol engineering technologies, including the use of ultra-high throughput microfluidic screening platforms, like droplet-based optical Pol sorting [311], have enabled the discovery of XNA Pols with activities approaching their natural counterparts. One exciting example in this area is a TNA Pol called 10–92 (Fig. 11) that can achieve a catalytic rate of ∼1 nts^−1^ and > 99% fidelity [312]. An X-ray crystal structure of the catalytically active conformation reveals large conformational changes relative to its closest natural homolog, indicating that the evolutionary distance required to transition natural DNA Pols into highly specialized XNA Pols may be greater than previously thought [312]. As work in this area continues, it will be interesting to see how closely Pols engineering efforts can mimic the activity of natural enzymes, and the degree of structural change required to achieve such levels of activity.

## XNA therapeutics

From 1998 to 2025, the United States Food and Drug Administration (FDA) approved 25 oligonucleotide therapeutics. These include seven antisense oligonucleotides, five splice-switching oligonucleotides, two aptamers, six small interfering RNAs (siRNAs), two vaccine adjuvants, two mRNA COVID-19 vaccines, and one telomerase inhibitor [214]. Common chemical modifications found in these drugs include substitution of the 2′ hydroxyl position of ribose with fluorine (2′F RNA), methoxy (2′-*O*Me RNA), 2′-*O*-(2-methoxyethyl) (MOE RNA) groups, phosphorothioate linkages (PS), and nucleobase substitutions, such 5-methyl cytosine and N1-methyl pseudouridine (mRNA vaccines). Another XNA modification found in oligonucleotide therapeutics currently in clinical use is the phosphorodiamidate morpholino (PMO) analog. It is used exclusively in splice-switching oligonucleotides (SSOs) targeting different exons of dystrophin mRNA for the treatment of Duchenne muscular dystrophy (DMD), specifically in the drugs Exondys 51, Vyondys 53, Viltepso, and Amondys 45 [313, 314].

In some applications, achieving a strong pairing between nucleotides may not always be desirable. For example, a comparison of *in vivo* RNAi silencing potencies targeting factor VII between siRNAs containing 2′-F RNA, 2′-*O*Me RNA, MOE-RNA, and LNA pyrimidines showed that 2′-F RNA was the most effective analog [315], illustrating the idiosyncrasies of drug discovery. Interestingly, while the 2′-F modification is highly effective, it provides less of a boost to thermal stability compared to the MOE-RNA and LNA analogs [315]. Thermal rebalancing in critical regions of siRNA duplexes and the strategic introduction of destabilizing modifications are key concepts in the design of RNAi therapeutics. These strategies have led to breakthroughs, such as the use of chiral acyclic (*S*)-GNA, which have helped mitigate off-target effects [214].

**Figure 12.**
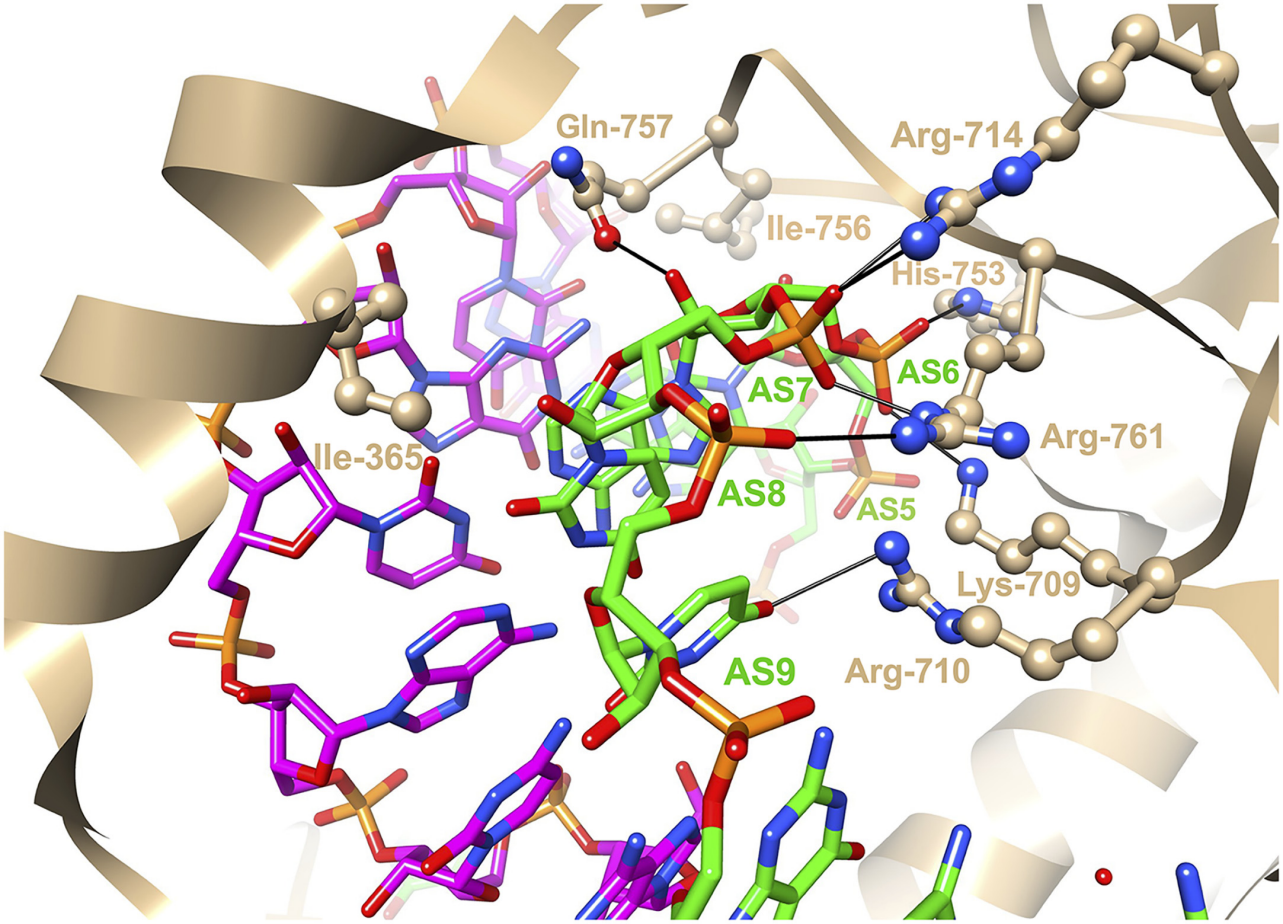
View across the minor groove of the siRNA antisense-/sense-strand duplex bound to Ago2 [318]; PDB ID 4w5t). Carbon atoms of the antisense and sense strands are colored in green and magenta, respectively. Selected Ago2 side chains and antisense siRNA residues are labeled, and H-bonds and salt bridges are indicated with thin solid lines.

Further insights can be gained from crystal structures of human Argonaute 2 (Ago2) in complex with miRNA single strands or siRNA duplexes. These structures reveal a strong kink in the antisense siRNA near the 3′-end of the seed region, between residues AS6 and AS7 [316, 317]. The distance between adjacent phosphorus atoms in the antisense siRNA at the kink is as short as 5.5 Å. Three Ago2 side chains—Ile-365, Gln-757, and Ile-756—straddle the minor groove of the seed region. The glutamine side chain forms a H-bond with the 2′ hydroxyl group of AS6 (Fig. 12). The electrostatic repulsion caused by closely spaced phosphates at the kink is offset by a cluster of three arginine residues, along with lysine and histidine, which gather around the AS6 to AS8 phosphate moieties.

**Figure 13.**
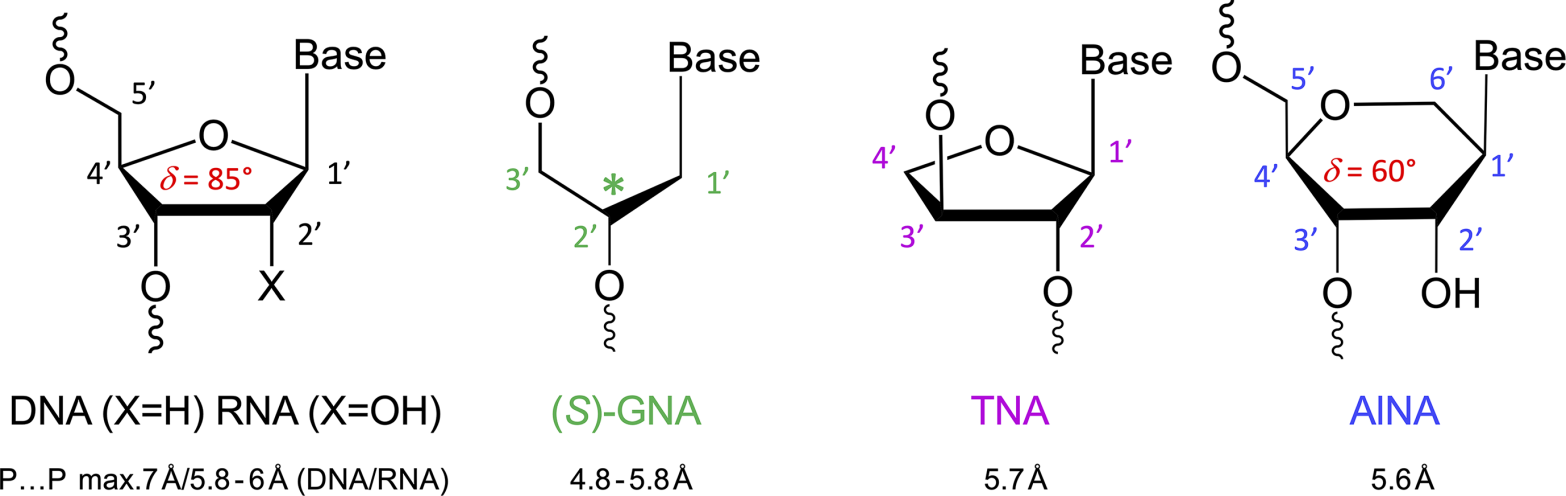
Structures of DNA, RNA, (*S*)-GNA, TNA, and AltNA. Distances below the structures correspond to average spacings between phosphorus atoms of 5′- and 3′-phosphates (B-DNA, A-RNA, and AltNA) and 3′- and 2′-phosphates (GNA, TNA).

The kink in the RNA structure results in tighter spacing between adjacent phosphates, a feature that can be mimicked by (*S*)-GNA and TNA (Table 1, Fig. 13). Both XNAs have only five bonds between phosphates in their backbones, compared to six in standard DNA and RNA. This naturally matches the 5.5 Å spacing observed between phosphates in kinked antisense siRNA bound to Ago2. (*S*)-GNA and TNA can cross-pair with RNA but incorporating single nucleotides or base pairs into RNA results in significant destabilization of pairing.

When these XNAs were walked along both antisense and sense strand siRNAs, *in vitro* activity measurements showed that both (*S*)-GNA and TNA were tolerated at several sites [316, 319]. In particular, substitutions at positions AS6 and AS7 (Fig. 12) led to increased potency. This effect is thought to stem from local softening of pairing constraints and preorganization of the antisense siRNA to favor the kinked conformation. AltNA, which also features closer spacing between adjacent phosphates (Fig. 13), was similarly tested. When AltNA residues were placed at the kink site, beneficial effects on activity were observed [320]. The importance of preorganizing the antisense siRNA for the kinked conformation in enhancing activity was further confirmed by the favorable activity of siRNA incorporating 2′-deoxy-2′-α-F-2′-β-C-methyl (gem-2′-F/Me) nucleotides at positions AS6 or AS7 [321]. While this modification does not reduce phosphate spacing, computational models suggest that the methyl group of gem-2′-F/Me pushes away the neighboring nucleobase, triggering a roll-bend that resembles the kinked conformation seen with native RNA.

Besides the beneficial effects for potency and safety of therapeutic siRNA candidates containing the abovementioned XNA residues, it is worth mentioning that placement of a 2′-5′ RNA modification at AS7 was shown to significantly reduce siRNA seed-mediated binding to off-target transcripts while maintaining on-target activity [172]. Earlier investigations directed at the effects of 2′-5′ linkages inside siRNA on RNAi activity had established that such modifications were generally well tolerated inside the sense strand but only at a few positions in the antisense strand [322]. Thus, 5′-phosphorylation of the siRNA antisense strand was minimally affected by the presence of a 2′-5′ linkage between AS1 and AS2. However, several modifications inside the antisense strand negatively affected siRNA loading into Ago2. Of note is the finding that 2′-5′ linkages abrogated the immune-stimulatory effects of modified siRNAs.

Clinically, the beneficial effects of XNAs at certain positions of antisense siRNA observed *in vitro* translated into increased potencies *in vivo*. Notably, the inclusion of (*S*)-GNA and TNA at position AS7 mitigated off-target effects. Although these two XNAs share similarities, they do not pair with one another [323]. A unique feature of the (*S*)-GNA duplex is its adoption of a right-handed A-form-like backbone conformation, combined with an inverted base pair orientation within the stack [214, 215]. GNA does not align with RNA when incorporated into the siRNA duplex, retaining its flipped nucleobase orientation opposite the RNA pairing partner. The destabilization of G:C pairs, relative to A:U pairs, can be offset by substituting iso-C or iso-G GNA residues opposite ribo-G or ribo-C, respectively [102]. The GalNAc-conjugate enhanced stability chemistry (ESC+), which incorporates (*S*)-GNA at position AS7 for liver delivery, has significantly improved potency and clinical safety due to seed pairing destabilization and conformational preorganization [172]. Importantly, this effect cannot be replicated by simply incorporating 2′-deoxyribonucleotides into the seed region to modulate thermal stability.

Two siRNAs in clinical development—ALN-AAT, that targets the Alpha-1 antitrypsin gene, and ALN-HBV, that targets Hepatitis B virus—were initially found to cause transient, asymptomatic liver enzyme elevations in a dose-dependent manner in patients [214, 215]. Similar elevations had been observed in preclinical rodent studies, but the hepatotoxicity was attributed to specific sequences rather than modification chemistry. The ESC+ approach, which mitigates off-target effects and improves safety, prompted the redesign of ALN-AAT and ALN-HBV by incorporating a single (*S*)-GNA residue at the AS7 seed region (ALN-AAT02 and ALN-HBV02, respectively) [214]. These modified siRNAs were evaluated in a Phase I study with healthy volunteers, demonstrating improved preclinical safety profiles. Neither siRNA led to elevated liver enzyme levels at the highest dose, confirming the benefits of the ESC+ design approach and the advantage of incorporating XNA at specific sites.

Several ESC+ candidate siRNAs with GNA incorporation are currently in clinical development, with ALN-HBV02 having advanced to Phase II trials. Currently, GNA has only been applied to RNAi therapeutics. However, we expect that more candidate therapeutics using various modalities that target a plethora of human diseases [324–327] and modified with XNAs beyond those in already approved drugs will enter the clinic in the coming years.

## Conclusions

XNAs, defined as sugar-modified nucleic acids, were first introduced in the context of synthetic biology. Their nomenclature spans the entire alphabet of 26 letters with their sugar moiety defining their position in the alphabet. XNAs can be categorized into those that hybridize with natural nucleic acids and those that do not. Structural parameters such as sugar puckering, inclination angle, and helical twist may help further classify them into subgroups of related systems.

XNAs that cross-pair with DNA and RNA have been extensively studied for their potential in therapeutic and diagnostic applications, particularly in interactions with cellular biology [328]. This field has greatly advanced with the clinical use of antisense, splice-switching, siRNA and aptamer drugs featuring chemically modified RNA mimics like 2′-*O*Me, 2′-F, 2′-MOE, and PMO which have been retrospectively classified as XNAs. Among these, TNA, HNA, and PNA hold a unique position, as they were the first XNAs designed without using DNA or RNA chemistry as a blueprint, yet they hybridize with natural nucleic acids. Their discovery was influenced by considerations of the origin of life and the structural folding of other bioorganic molecules, such as carbohydrates and peptides.

Representative orthogonal XNAs include pRNA, XyloNA, and L-DNA, whereby the latter has been utilized as a genetic system for aptamer development. The field of XNA research remains in its early stages and presents additional complexity, as it requires selecting XNAs that function as agonists *in vivo* rather than antagonists. A promising development is the discovery of key tools such as XNA Pols and ligases, although we are still far from an orthogonal replication system capable of evolving and encoding new functions within a cell. However, it is noteworthy that faithful transcription using a chemically synthesized, mirror-image T7 RNA Pol of L-RNA 5S, 16S, and 23S rRNAs has been achieved from L-DNA genes [329].

While therapeutics and diagnostics constitute important fields for XNA applications, these nucleic acid analogs are also used in other practical applications. For examples in bio- and nanotechnology, see e.g. CeNA, FANA, and HNA nanostructures visualized by TEM [330], FANA nanostructures as stable carriers for cellular delivery [331], TNA-shielded DNA nanostructures [332], and HNA as glue triggering the controlled formation of bacterial tissues [333]. In the realm of drug discovery, complementary PNA oligonucleotides mediated assembly of a supramolecular inhibitor of thrombin whose action was reversed with an antidote oligo [334].

## Data Availability

Table 1 is limited to XNA systems that have been incorporated into oligonucleotides and is currently maintained at https://chaputlab.com/xna-alphabet/.
